# Genomic view of the diversity and functional role of archaea and bacteria in the skeleton of the reef-building corals *Porites lutea* and *Isopora palifera*

**DOI:** 10.1093/gigascience/giac127

**Published:** 2023-01-23

**Authors:** Kshitij Tandon, Francesco Ricci, Joana Costa, Mónica Medina, Michael Kühl, Linda L Blackall, Heroen Verbruggen

**Affiliations:** School of BioSciences, University of Melbourne, Parkville 3010, Australia; School of BioSciences, University of Melbourne, Parkville 3010, Australia; Biological, Earth and Environmental Sciences, The University of New South Wales, Kensington, NSW 2052, Australia; School of BioSciences, University of Melbourne, Parkville 3010, Australia; Department of Biology, Pennsylvania State University, University Park, PA 16802, USA; Marine Biological Section, Department of Biology, University of Copenhagen, DK-3000 Helsingør, Denmark; School of BioSciences, University of Melbourne, Parkville 3010, Australia; School of BioSciences, University of Melbourne, Parkville 3010, Australia

## Abstract

At present, our knowledge on the compartmentalization of coral holobiont microbiomes is highly skewed toward the millimeter-thin coral tissue, leaving the diverse coral skeleton microbiome underexplored. Here, we present a genome-centric view of the skeleton of the reef-building corals *Porites lutea* and *Isopora palifera*, through a compendium of ∼400 high-quality bacterial and archaeal metagenome-assembled genomes (MAGs), spanning 34 phyla and 57 classes. Skeletal microbiomes harbored a diverse array of stress response genes, including dimethylsulfoniopropionate synthesis (*dsy*B) and metabolism (DMSP lyase). Furthermore, skeletal MAGs encoded an average of 22 ± 15 genes in *P. lutea* and 28 ± 23 in *I. palifera* with eukaryotic-like motifs thought to be involved in maintaining host association. We provide comprehensive insights into the putative functional role of the skeletal microbiome on key metabolic processes such as nitrogen fixation, dissimilatory and assimilatory nitrate, and sulfate reduction. Our study provides critical genomic resources for a better understanding of the coral skeletal microbiome and its role in holobiont functioning.

## Introduction

Symbiont-bearing, reef-building corals harbor diverse microbiomes, forming a multispecies consortium termed the coral holobiont [1]. Much like other multicellular organisms, corals rely on their microbiome for health and functioning [2,3]. A rapid decline in coral reefs across the globe has shifted the focus to characterizing the functional role of coral-associated bacteria, unarguably the most diverse members of the coral holobiont. Bacteria have the potential for developing effective assisted evolution strategies such as coral probiotics [4,5] and microbiome manipulation [6] to protect coral reefs. Recent studies showed that coral-associated bacteria play significant roles (e.g., in nutrient recycling [7–9] and protection against pathogens [7] that can govern coral health). Bacterial community composition profiles also serve as indicators of coral health exhibiting shifts to less diverse microbiomes with a stronger predominance of pathogens during dysbiosis [10–12]. However, it is important to note that most of our present knowledge on coral microbiomes and their role in coral holobiont fitness and health stems from investigations of the millimeter-thick coral tissue layer [13]. This layer is spread over a voluminous calcium carbonate structure (i.e., the coral skeleton), which harbors endolithic microorganisms. This microhabitat is often neglected in coral microbiome research but represents a key ecological niche for microorganisms in the coral holobiont [14].

The bulk of the coral skeleton, except the tissue–skeleton interface and upper millimeters of the skeleton in shallow-water corals, receives low irradiance and exhibits a broader array of microenvironmental dynamics than the coral tissue [3]. Metabarcoding surveys have revealed vast microbial and microeukaryotic diversity, including archaea [15, 16], bacteria [17–22], fungi [23–25], and protists such as endolithic green algae in the genus *Ostreobium* [17], showing that the skeletal microbiome differs significantly from that of other coral compartments [26]. Microboring, filamentous green algae (*Ostreobium* spp.) form conspicuous green bands in the skeletons of several coral species. *Ostreobium* can play an active role in both providing carbon substrates to coral hosts during thermal stress–induced bleaching and facilitating coral recovery [27–29]. Endolithic microbes have also been reported to actively participate in nutrient recycling and primary productivity [13, 14]. Functional characterization of complex microbial assemblages in the coral skeleton has mainly relied on selective amplification of target genes (e.g., *nifH* [30–32]), experimental approaches such as the acetylene reduction technique, and isotope labeling for probing N_2_ fixation and other metabolic activities [33, 34]. While such studies have yielded important insights into the coral skeleton niche, a genome-centric view of the coral skeletal microbiome and its functional potential remains elusive.

Whole-genome shotgun sequencing complemented with metagenome binning has been applied to recover genomes of dominant green-sulfur bacteria belonging to the genus *Prosthecochloris* in the skeleton of coral *Isopora palifera* [20,35]. These studies are the only shallow-depth genome-centric research conducted to date on the coral skeleton, with a combination of FISH-nanoSIMS (Fluorescence in situ hybridization-nanoscale secondary ion mass spectrometry) and the acetylene reduction assay to confirm the dinitrogen-fixing ability of dominant anaerobic phototrophs. A recent study used a combination of metabarcoding and gene- and genome-centric metagenomics to shed light on the role of the endolithic microbiome in coral bleaching susceptibility [36]. The limited insights into the broader functional potential of coral skeletal microbiomes hamper our ability to identify key roles of the skeleton microbiota within the coral holobiont.

To address these knowledge gaps, we applied a deep sequencing metagenomics approach to obtain metagenome-assembled genomes (MAGs) from bacteria and archaea residing in the skeletons of the 2 reef-building corals *Porites lutea* (NCBI:txid51062) and *I. palifera* (NCBI:txid105615). We further explored the potential of these microbiome members to provide essential functions to the coral holobiont in terms of engaging in symbiosis with the host, their ability to mitigate oxidative stress, and their role in biogeochemical nutrient cycling.

## Materials and Methods

### Sample collection and processing

Fragments from 5 individual healthy-looking colonies of *P. lutea* and *I. palifera* were each collected at low tide (<1 m depth) from the research zone of the Heron Island reef flat, central Great Barrier Reef (23°44′S, 151°91′E), in January 2020. The fragments were collected using a sterile hammer and chisel and were immediately placed in sterile ziplock polyethylene bags in seawater. Coral tissue was removed from the fragments using a Waterpik and sterile seawater. Coral fragments with only skeletons were snap-frozen by immersion in liquid nitrogen and stored at −80°C until processing.

### DNA isolation, library preparation, and whole metagenome sequencing

Total DNA was extracted using the DNeasy PowerSoil Pro Kit (Qiagen, Hilden, Germany) as per the manufacturer's protocol. Extracted DNA samples were sent to BGI Tech Solutions (Hong Kong) for library preparation and sequencing on individual lanes per sample using DNBSeq (2 × 150). On average, we obtained >327 million and >298 million read pairs for *P. lutea* and *I. palifera*, respectively.

### Read quality control, trimming, and removal of host-related reads

Paired-end reads were quality checked using FASTQC [37] and multiQC (RRID:SCR_014982) [38]. Reads were trimmed with trimmomatic v0.38 (RRID:SCR_011848) [39] with the following parameters: *HEADCROP:5 SLIDINGWINDOW:4:20 MINLEN:30*. Trimmed reads from 5 *P. lutea* samples were mapped to its draft genome downloaded from reefgenomics.org [40] using bowtie2 (RRID:SCR_016368) with default settings [41]. Unmapped paired-end reads were extracted using samtools v1.7 (RRID:SCR_002105) [42]. Two samples, PL23b_i and PL25b_i, had 48.19% and 63.66% reads mapping to the *P. lutea* genome, and an additional full lane of sequencing was performed for them and processed with the same specifications. Only paired-end unmapped reads were used for *de novo* metagenome assembly. Paired-end trimmed reads from *I. palifera* metagenome samples were directly assembled as the host genome is not available.

### Metagenome assembly, binning, and dereplication

Metagenome assembly was performed on individual samples using MegaHIT v1.2.9 (RRID:SCR_018551) [43] with *k*-mers 33, 55, 77, and 99 and a minimum contig length of 1,000. Resultant contigs per sample were binned using Concoct v1.0.0 [44], Maxbin2 v2.2.6 [45], and Metabat2 v2.12.1 (RRID:SCR_019134) [46] as implemented in MetaWrap v1.3.2 [47]. One sample of *I. palifera* (IP31a_i) yielded no bins from Maxbin2, and this sample was additionally binned using Metabat1 [48]. Obtained bins were then refined using the bin_refinement module of MetaWrap with parameters *completeness > = 50%* and *contamination < = 10%*. Refined bins from all the samples were pooled and dereplicated using dRep v3.0.0 [49] using default parameters. CheckM v1.0.12 (RRID:SCR_016646) [50] was used to estimate the completeness and contamination statistics of dereplicated bins; only bins with at least 80% completeness and less than 10% contamination were selected for downstream analysis. Bins were subjected to CAT and BAT v5.2.3 [51] to identify misbinned contigs based on taxonomic affiliation in a bin using default parameters. Contigs annotated as Eukaryota were removed from the bins using a custom python script available on Figshare [52]. Completeness and contamination statistics were again evaluated as above. We categorized the bins into high quality (completeness >80% and contamination <10%) and medium to low quality (completeness >50% to <80% and contamination <10%) based on CheckM completeness and contamination statistics. Only high-quality bins were used for downstream processing and were called MAGs.

### Taxonomic assignment and relative coverage of MAGs

Taxonomic assignment of each dereplicated and CAT- and BAT-corrected bin was performed based on the Genome Taxonomy Database release 202 using the *de_novo_wf* approach implemented in GTDB-Tk [53]. GTDB-Tk classifies MAGs by placing them in a referenced tree inferred using a set of 120 bacterial and 122 archaeal concatenated gene markers using a combination of FastANI (RRID:SCR_021091) [54] and pplacer (RRID:SCR_004737) [55]. We mapped each coral species–specific metagenomic trimmed paired-read set to coral species–specific MAGs using BBMap (RRID:SCR_016965) [56], which generates coverage information using *pileup*. To calculate the relative coverage as a proxy for abundance across the samples, we calculated the average coverage per contig per MAGs and converted it to a relative coverage profile to represent the genome coverage per sample. Stacked barplots were generated in R v4.0.2 [57] using ggplot2 (RRID:SCR_014601) [58] to represent relative-read coverage of MAGs per coral colony.

### Phylogenetic tree building and visualization

Archaeal and bacterial phylogenetic trees were constructed by providing respective concatenated marker gene alignments generated by GTDB-Tk to IQ-TREE v1.6.1 [59] with LG+G selected as the model and 1,000 ultrafast bootstraps. Phylogenetic trees with genome statistics across microbial lineages and distribution of genes of interest and functional pathways (see details below) were visualized using the iTOL v6 [60].

### Gene prediction, annotation, and metabolic potential

Prodigal v2.6.3 (RRID:SCR_011936) [61] implemented in Prokka v1.14.5 (RRID:SCR_014732) [62] was used for gene prediction. Predicted genes per MAGs were then provided to Interproscan v5.53.87 (RRID:SCR_005829) [63] to search for protein family (Pfam) ids (*-appl Pfam*), with -evalue cutoff of -1e-5. Unique hits from filtered output were searched for genes of interest, including eukaryotic-like proteins (ELPs): WD40 repeats proteins (WD40) (PF00400 and PF07676), ankyrin repeat proteins (ARP) (PF00023 and PF13857), HEAT repeat proteins (HEAT) (PF13646), tetratricopeptide repeat (TPR) (PF00515, PF07719, PF09976, PF13174, PF13181, PF13371, PF13374, PF13424, PF13428, PF13429, PF13431, PF13432, PF14559, PF14561, and PF16918), nitrogen fixation (*nif*H) (PF00142), dimethylsulfoniopropionate metabolism (DMSP) synthesis (*dsy*B) (PF00891 and PF16864) and catabolism (DMSP_lyase) (PF16867), superoxide dismutase (SOD) (PF00080, PF00081, and PF02777), catalase (PF00199), and ammonia oxidation (AmoA) (PF12942). METABOLIC-G, implemented in METABOLIC [64], was used for annotation of KEGG pathways to determine the functional potential of MAGs using the following parameters: *-m-cutoff 0.50*. Results from METABOLIC-G on a per MAG level were parsed and collated using the CSV/TSV tool kit [65]. Collated output was combined as a matrix and used as input to EnrichM [66] *classify* workflow for calculating the completeness of predicted KEGG modules. KEGG modules with >75% completeness for nitrogen metabolism, sulfur metabolism, and anoxygenic photosynthesis in any samples were plotted as a heatmap using pheatmap [67] in R as well as visualized in iTOL v6.

## Results

### Sequencing overview, *P. lutea* and *I. palifera* skeletal microbiome

**Figure 1:**
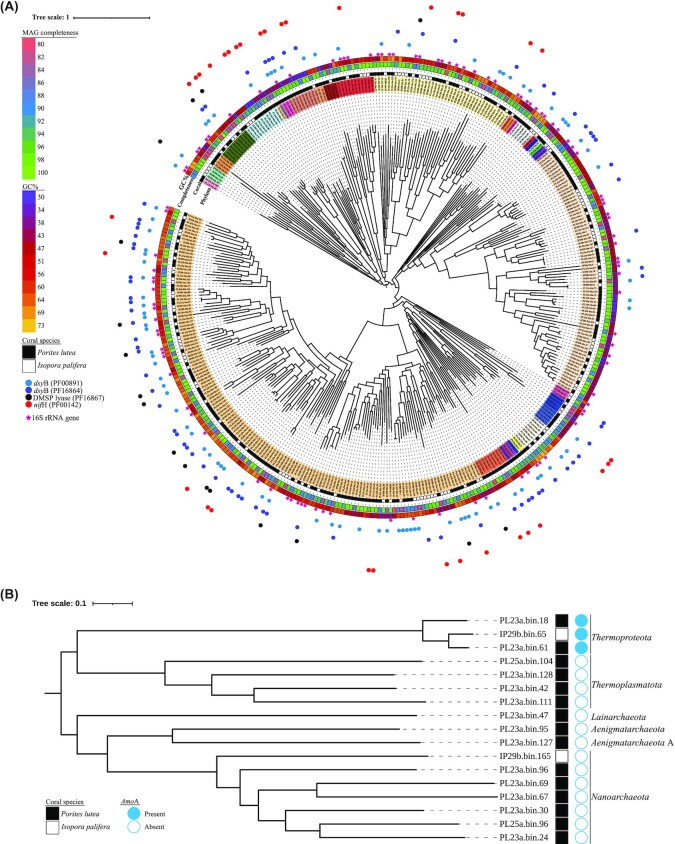
Phylogenetic trees of metagenome-assembled genomes (MAGs) recovered from *P. lutea* and *I. palifera* skeleton. (A) In total, 376 bacterial MAGs, with genome completeness, GC content, and genes of interest and (B) 17 archaeal MAGs with the presence of ammonia oxidizing gene *AmoA*. The phylogenetic tree was constructed using a concatenated alignment of 120 bacterial and 122 archaeal marker genes, respectively. Taxonomic annotation of bacterial MAGs (innermost circle): *Proteobacteria*,*Desulfobacterota*, DSWW01, *Desulfobacteriota*_F, *Nitrospinota*, *Bdellovibrionota*, *Desulfobacterota*_B, *Myxococcota*, SAR324, *Bdellovibrionota*, *Acidibacteriota*, *Bacteroidota*, *Calditrichota*, SM23-31, AABM5-125-54, *Marinosomatota*, Zixibacteria, Gemmatimondota, *Fibriobacterota*, *Elusimicrobiota*, *Omnitrophota*,*Sumerlaeota*,*Planctomycetota*,*Verrucomicrobiota*,*Chlamydiota*,*Spirochaetota*,*Firmicutes*_A,*Firmicutes*,*Fimicutes*_H,*Firmicutes*_G,*Cyanobacteria*,*Chloroflexota*,*Patescibacteria*,*Actinobacteriota*,*Bipolaricaulota*.

We sequenced a total of 1.6383 billion read pairs for *P. lutea* (3.39%–63.66% host) and 1.2952 billion read pairs for *I. palifera* samples (Supplementary Data File). We obtained 250 high-quality MAGs from *P. lutea* (average completeness ± standard deviation: 92.89% ± 5.53% and contamination: 2.22% ± 1.72%) and 143 from *I. palifera* (average completeness: 93.89% ± 5.46% and contamination: 1.91% ± 1.61%) (Supplementary Figs. S1 and S2). Of the 250 *P. lutea* MAGs, 235 were bacterial and 15 archaeal, and 141 of *I. palifera* 143 MAGs were bacterial and only 2 archaeal, based on GTDB-tk classification (Fig. 1A, B). A total of 113 MAGs (69 *P. lutea* and 44 *I. palifera*) had at least 1 copy of the 16S ribosomal RNA gene predicted in them (Fig. 1A, B; Supplementary Data File).

These MAGs spanned the vast majority of microbial lineages (34 phyla and 57 classes) in the coral skeleton (Fig. 1, Supplementary Data File), including bacteria from phyla *Proteobacteria* (147 MAGs), *Bacteroidota* (75), *Planctomycetota* (42), *Desulfobacterota* (12, including lineages B and F), *Firmicutes* (12, including lineages A, F, G, and H), *Cyanobacteria* (11), *Verrucomicrobiota* (11), *Chloroflexota* (10), *Myxococcota* (9), *Gemmatimonadota* (5), *Bdellovibrionota* (5), *Actinobacteriota* (4), *Chlamydiota* (4), *Patescibacteria* (4), SAR324 (4), *Acidobacteriota* (3), *Spirochaetota* (3), *Bipolaricaulota* (2), *Calditrichota* (2), AABM5-125-24 (1), DSWW01 (1), *Elusimicrobiota* (1), *Fibrobacterota* (1), *Marinisomatota* (1), *Nitrospinota* (1), *Omnitrophota* (1), SM23-31 (1), *Sumerlaeota* (1), *Zixibacteria* (1) and archeal phyla *Nanoarchaeota* (7), *Thermoplasmatota* (4), *Thermoproteota* (3) *Aenigmatarchaeota* (2, including lineage A), and *Iainarchaeota* (1).

### *P. lutea* and *I. palifera* harbor different skeletal microbiome

Comparing microbial communities recovered from MAGs that meet completeness (≥90%) and contamination (≤10%) thresholds, and basing our results on the presence and absence of MAGs from the 2 coral species, we identified some MAGs (*Actinobacteriota*,*Calditrichota*,*Sumerlaeota*, and *Zixibacter*) to be unique to *I. palifera* and some other MAGs (AABM5-125-24, *Bipolaricaulota*,*Desulfobacterota*,*DSWW01*,*Elusimicrobiota*,*Fibrobacterota*,*Firmicutes*,*Marinisomatota*,*Nitrospinota*,*Omnitrophota*,*Patesibacteria*, SAR324, and SM23-31) to be unique to *P. lutea*. We recovered 1 archaeal MAG each of *Thermoproteota* and *Nanoarchaeota* from *I. palifera* metagenomes, whereas *Iainarchaeota*, *Aenigmatarchaeota*, and *Thermoplasmatota* MAGs were recovered from *P. lutea* metagenomes.

MAGs recovered from *P. lutea* were differentially abundant among colonies, whereas the relative abundance of MAGs appeared stable among the colonies of *I. palifera* (Supplementary Fig. S3). *P. lutea* skeletal samples were dominated by MAGs from bacterial classes *Alphaproteobacteria*,*Vampirovibrionia*, and *Planctomycetes* and 1 sample (PL25b) was also dominated by archaeal phyla *Thermoproteota* (Supplementary Fig. S3). In contrast, *I. palifera* skeletal samples were dominated by MAGs from bacterial classes *Bacteroidia*,*Cyanobacteria*,*Anaerolineae*, and *Polygania*, with 1 colony (IP29b) harboring a relatively high abundance (45.76%) of *Cyanobacteria* MAG (IP29b_bin.176) (Supplementary Fig. S3).

### Skeletal bacteria show the potential to engage in symbiosis with eukaryotes

**Figure 2:**
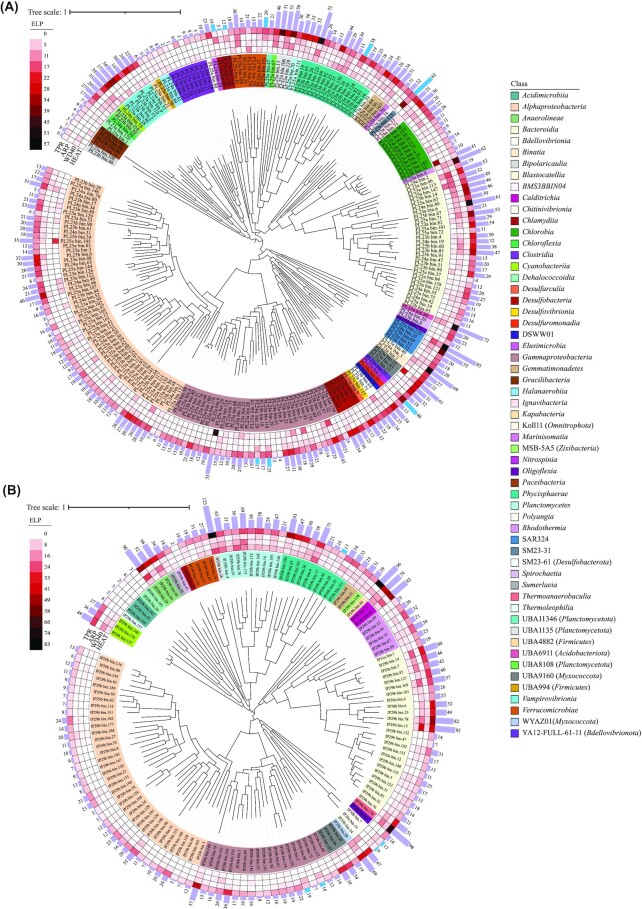
Phylogenetic tree of bacterial MAGs with representation of different categories of ELPs from (A) *P. lutea* and (B) *I. palifera*. From inner to outer, innermost circle represents MAGs color-coded at bacterial class level, heatmap represents different ELP categories (inner to outer: HEAT repeats; WD domain repeats, WD40; ankyrin repeats, ARPs; and tetratricopeptide repeats, TPRs), and barplot represents total ELP counts in MAGs. Bars colored in “sky blue” represent MAGs that devote >0.2% of total genes to ARPs, suggesting a potentially host-associated lifestyle. Detailed information about these MAGs and distribution of ELP protein families is available in the Supplementary Data File.

Microorganisms use ELPs to communicate with their hosts and other eukaryotes. Recovered MAGs on average encoded 0.56% ± 0.31% (*P. lutea*) and 0.61% ± 0.38% (*I. palifera*) ELPs per genome. MAGs had a broader range of ELPs, including WD or β-transducin repeats of 40 amino acids often terminating in tryptophan-aspartate dipeptide (WD40), TPRs, ARPs, and HEAT repeats, a set of 4 proteins first identified to contain this repeat motif (Huntington, elongation factor 3, subunit A of phosphatase 2A, and signallng kinase TOR1). The most abundant group of ELPs in MAGs from *P. lutea* and *I. palifera* included TPRs (*P. lutea:* TPR_16, Pfam: PF13432, avg. proteins: 3.88 ± 3.75; *I. palifera:* TPR_12: Pfam: PF13424, avg. proteins: 4.94 ± 8.27) (Fig. 2A, B). MAGs harbored relatively low numbers of WD40 (*P. lutea:* 2.65 ± 4.06; *I. palifera:*4.68 ± 6.32) and HEAT repeat proteins (*P. lutea:* 2.65 ± 5.04; *I. palifera:* 3.28 ± 4.77), with the highest count in a MAG from Candidate phylum SM23-31 (37 WD40 repeat proteins) in *P. lutea* (PL23a_bin.125) and a MAG from class *Bacteroidia* (39 WD40 repeat proteins) in *I. palifera* (IP29b_bin.15) (Fig. 2A, B). A MAG belonging to class UBA1135 (phylum: *Planctomycetes*) harbored 47 and 25 HEAT repeat proteins in *P. lutea* (PL25a_bin.29) and *I. palifera* (IP29b_bin.26), respectively (Supplementary Data File). ARPs were the least abundant ELPs in the MAGs (*P. lutea:* 2.09 ± 2.6; *I. palifera:* 2.58 ± 2.25). Out of 235 bacterial MAGs from *P. lutea*, no ARPs were identified in 72 MAGs, 63 MAGs had only 1 ARP, and there were 9 MAGs encoding more than 10 copies of ARP. In contrast, out of 141 bacterial MAGs from *I. palifera*, 23 had none, 36 MAGs had only 1 copy, and 2 MAGs had 10 ARPs (Supplementary Data File). Microbes are considered host associated if they devote more than 0.2% of their total gene repertoire to ARPs [68]. Keeping this conservative threshold as identified earlier, we identified only 10 MAGs belonging to 6 bacterial phyla from *P. lutea* and 5 MAGs from 3 phyla from *I. palifera* meeting this criterion (Fig. 2A, B). Further, all 3 *Chlamydia* MAGs from *P. lutea* and 2 *Bdellovibrionia* MAGs from *I. palifera* encoded >0.2% ARPs.

### The skeletal microbiome harbors an array of oxidative stress alleviators

Approximately half of the *P. lutea* bacterial MAGs (114) had at least 1 copy of the *dsy*B (PF00891, PF16864) gene, conferring the ability to synthesize DMSP, and 13 MAGs had at least 1 copy of the *DMSP_lyase* (PF16867) gene able to metabolize DMSP to other potent antioxidants (Fig. 1A). Although the ability to synthesize DMSP was identified in 48.5% of MAGs, only 8 MAGs had at least 1 copy of both *dsy*B and DMSP_lyase genes (Fig. 1A), with 7 of these belonging to the class *Alphaproteobacteria* and 1 to *Gammaproteobacteria* (Supplementary Data File). The catalase gene (PF00199) was identified in 13 MAGs. At least 1 copy of the superoxide dismutase, *SOD*, gene (including SODC [PF00080] and *SOD_Fe_N* [PF00081]) was identified in 94 bacterial MAGs. In contrast, out of 141 bacterial *I. palifera* MAGs, 58 had at least 1 copy of the *dsy*B gene, and 16 MAGs had a copy of *DMSP_lyase* (Fig. 1A). Further, only 13 MAGs belonging to class *Alphaproteobacteria* (11 MAGs), *Anaerolineae* (1 MAG), and *Acidimicrobia* had at least a copy of *dsy*B and *DMSP_lyase* genes (Supplementary Data File). SOD genes were annotated in 74 MAGs, and catalase genes were identified in 5 MAGs only.

### Skeletal archaea and bacteria engage in nitrogen and sulfur metabolism

We identified that 87 *P. lutea* and 45 *I. palifera* MAGs harbor the potential to fix nitrogen with at least 1 copy of the *nif*H gene (PF00142) (Fig. 1A). Ammonia oxidation, *the Amo*A gene, was identified in 2 and 1 archaeal MAGs from *P. lutea* and *I. palifera*, respectively (Fig. 1B).

**Figure 3:**
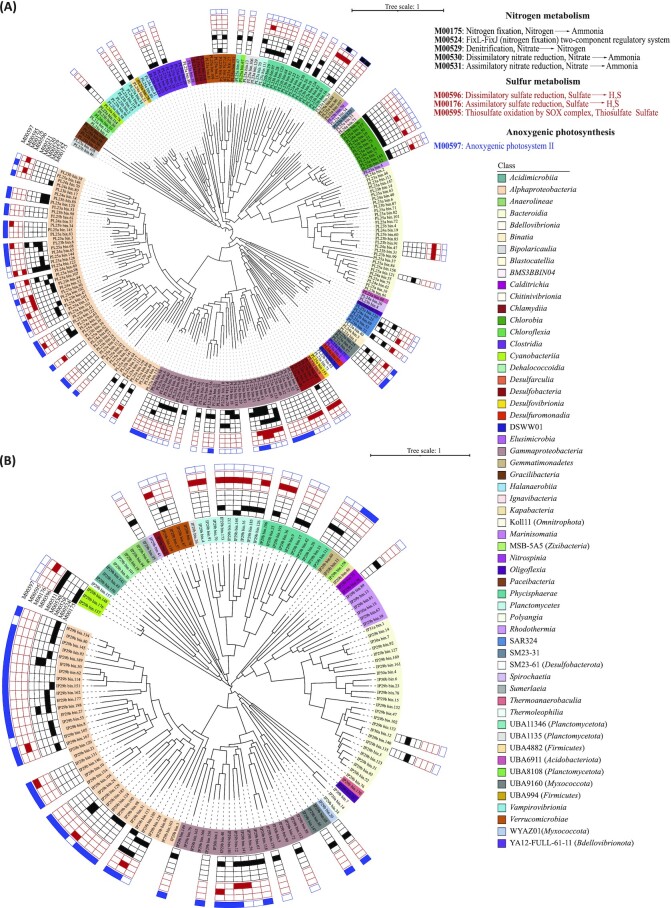
Phylogenetic tree of bacterial MAGs representing functional pathways based on nitrogen, sulfur, and anoxygenic photosynthesis KEGG modules in (A) *P. lutea* and (B) *I. palifera*. From inner to outer ring, innermost circle represents MAGs color-coded at bacterial class level. Heatmap represents different KEGG modules (from inner to outer) for nitrogen metabolism (M000175, M00524, M00529, M00530, and M00531), sulfur metabolism (M00596, M00176, and M00595), and anoxygenic photosynthesis (M00597). MAGs with at least 1 KEGG module 100% complete are shown here.

We analyzed the processes involved in nitrogen cycling, including nitrification, denitrification, nitrogen fixation, and assimilatory and dissimilatory nitrate reduction, to obtain comprehensive insights and understanding of nitrogen metabolism by the members of the coral skeleton microbiome. The nitrogen fixation module (M00175) was identified as complete in several MAGs, with 10 MAGs of *Chlorobia* and 3 of *Clostridia* encoding complete nitrogen fixation modules in *P. lutea* (Fig. 3A) and 2 *Cyanobacteria* MAGs, 4 *Alphaproteobacteria* MAGs, and 1 *Planctomycetes* MAG harboring the potential to fix nitrogen in *I. palifera* (Fig. 3A). Interestingly, the oxygen-dependent regulatory nitrogen fixation module (M00524) mediated by FixL-FixJ genes was also complete in several MAGs belonging to *Alphaproteobacteria*,*Gammaproteobacteria* (order: *Pseudomonadales*, UBA4575, *Xanthomonadales*, *Woeseiales*, HTCC5015, DSM-100 275, and *Chromatiales*),*Phycisphaerae*, and *Planctomycetes* in 2 coral species (Fig. 3B, Supplementary Data File). The dissimilatory nitrate reduction module (M00530), producing ammonia from nitrate, was complete in MAGs spanning different bacterial classes in the 2 coral species (Fig. 3A, B). However, assimilatory nitrate reduction (M00531) ability was poorly represented, with only MAGs from *Cyanobacteria* and *Alphaproteobacteria* encoding the complete module. The denitrification module was complete in 1 MAG each belonging to *Gammaproteobacteria* in *P. lutea* (Fig. 3A) and *Alphaproteobacteria*, *Anaerolineae*, and *Bacteroidia* in *I. palifera* (Fig. 3B).

In oceans, sulfur is available as inorganic sulfate that can be assimilated by microbes into organic compounds. We searched for the ability of coral skeletal microbes to assimilate inorganic sulfur and use it to produce organic compounds as well as for energy-yielding purposes. We identified MAGs of sulfate-reducing bacteria (SRB), including members of *Desulfobacteria*, *Desulfarculia*, and SAR324 encoding the complete dissimilatory sulfate reduction module (M00596), along with a few MAGs belonging to *Gammaproteobacteria*,*Chlorobia*, and *Alphaproteobacteria* in *P. lutea* (Fig. 3A), whereas in *I. palifera*, only 2 *Gammaproteobacteria* MAGs had the complete module (Fig. 3B). We found complete assimilatory sulfate reduction modules in MAGs from several lineages, including *Alphaproteobacteria*,*Bacterodia*,*Binatia*,*Gammaproteobacteria*,*Phycisphaerae*,*Planktomycetes*, and *Verrucomicrobiae* from the 2 coral species (Fig. 3A, B). Further, complete anoxygenic photosystem II module (M00597) was identified in several MAGs belonging to purple sulfur and purple nonsulfur bacteria from different classes, including *Alphaproteobacteria*,*Anaerolineae*,*Gammaproteobacteria*,*Gemmatimonadetes*, and others in both coral species (Fig. 3A, B). Bacteria harboring this module have the potential to use H_2_S produced by assimilatory and dissimilatory reduction of sulfate as a primary electron donor. Some of these above-described MAGs also harbored partially complete KEGG modules of interest (Supplementary Fig. S4).

## Discussion

With coral reefs under significant pressure across the globe due to climate change and other stressors derived from anthropogenic activities, coral microbiome research has recently been gaining a lot of traction for the development of coral probiotic and assisted evolution strategies, including microbiome manipulation and build-out of thermotolerant microbial symbionts to protect reefs [6, 8, 69–72]. Here, we describe a compendium of bacterial and archaeal high-quality MAGs recovered from skeletons of 2 dominant reef-forming coral species, *P. lutea* and *I. palifera*. Our results provide an unprecedented view of the coral skeletal microbiome, permitting more detailed discussion of the community composition, the ability of endolithic microbes to form symbiosis with eukaryotes within the coral skeleton, and the functional roles these endoliths can play in nutrient cycling and holobiont functioning.

### Genome-centric view of the skeletal microbiome

The biggest challenge in working with host microbiomes is the contamination from the host DNA, which is often compounded by the lack of host genome required to remove host-related sequencing reads. This was true in our study, with *P. lutea* skeletal samples showing varying proportions of host reads (Supplementary Table S1) and lack of *I. palifera* genome to account for host-related reads in the samples. Considering there was some coral tissue sequenced, it is reasonable to assume that some of the MAGs reported in this study might also not be exclusively found in the coral skeleton. In that context, it is important to note that there is no strict boundary between the coral tissue and the skeleton, and upper layers of the coral skeleton also harbor coral tissue–associated bacteria as reported in our earlier study [22]. Using deep metagenomic sequencing, we recovered a compendium of 376 bacterial and 17 archaeal high-quality MAGs from the skeleton of *P. lutea* and *I. palifera* corals (Fig. 1A, B). The community composition of recovered MAGs reflects on studies using marker gene surveys to profile the coral skeletal microbial community often dominated by members of classes *Alphaproteobacteria*,*Clostridia*, and *Chlorobia* for *P. lutea* and *Bacteroidia*,*Anaerolineae* (phylum: *Chloroflexota*), and *Chlorobia* for *I. palifera* [17–19, 21, 22, 73]. The community composition of MAGs recovered from *P. lutea* skeletons studied here was vastly different from 52 MAGs reported from *P. lutea* tissue in a recent study [74]. MAGs recovered from *P. lutea* tissue belonged to *Poribacteria*,*Actinobacteriota*, *Dadabacteria*, *Latescibacterota*, and UBP10, which were not recovered in our study. But we did recover MAGs belonging to the archaeal class *Nitrososphaeria* and a few bacterial classes. Further, we identified a similar MAG community composition, although with significantly more diversity of MAGs recovered in our study compared to a recent study using a genome-centric approach on coral skeleton [36]. In light of these comparisons, we provide an exhaustive collection of skeletal dominated coral-associated bacterial and archaeal MAGs.

### Skeletal microbiomes harbor an array of ELPs to form stable symbiosis with eukaryotes in the coral holobiont

The coral holobiont is highly complex with the presence of several microeukaryotes and a high microbial diversity. Corals and potentially these microeukaryotes rely on prokaryotic microbes for fulfilling their metabolic requirements. Therefore, these microbes must harbor the genetic machinery to interact with the host without eliciting the host's immune response, (e.g., by harboring proteins containing ELPs). Coral-associated bacteria harboring these ELPs also have the potential to interact with other microeukaryotes present in the coral skeleton, including endolithic microalgae (e.g., *Ostreobium*,*Phaeophila* [18], sponges, and corallimorphs), as well as endolithic fungi including *Ascomycota* and *Basidiomycota* [21, 23, 75], among others. Proteins containing these repeats are commonly associated with additional functional domains such as lipid metabolism and mediating ubiquitination; therefore, these are likely to engage host proteins directly [76]. Although ELPs have been prevalent in bacteria associated with marine invertebrates, including corals, the presence of different types of ELPs in bacterial genomes raises the question of their diverse roles and how one should weigh the importance of one type of ELP over others. A recent comprehensive study suggests that ELP abundance is determined by different factors [68]. ARP abundance is more related to the lifestyle of the bacteria, whereas TPR abundance is determined by phylogenetic history rather than lifestyle [68, 77].

Ankyrin repeats, which span 30 to 40 amino acids and exclusively function in mediating protein–protein interactions [78], are a well-characterized group of ELPs. In *Escherichia coli*, ankyrin repeat–containing genes, when expressed, were shown to help modulate phagocytosis by sponge amoebocytes, suggesting a possible mechanism by which symbionts can evade digestion from host cells and establish symbiosis [79]. Coral-associated bacteria have been reported to contain a wide array of ARPs, with high gene copies in tissue-associated bacteria, including *Endozoicomonas* [9], *Poribacteria* [74], and low dominance in *Vibrio* strains [80]. In the present study, members of diverse microbial lineages were identified to harbor ARPs ranging from 1 to 13 proteins in *P. lutea–*associated bacteria and 1 to 10 proteins in *I. palifera–*associated bacterial MAGs. Only a handful of MAGs devoted more than 0.2% of total proteins to ARPs in the current study, suggesting that only a few microbial symbionts that colonize the coral skeleton potentially have a strictly host-associated lifestyle and could be obligate symbionts. *Chlamydiae* are strictly intracellular and therefore intimately reliant on their hosts [81], and 3 high-quality *Chlamydiae* MAGs recovered from *P. lutea* colonies devoted more than 0.2% of their proteome to ARPs (Fig. 2A).

WD40 proteins are widespread in eukaryotes but are rare in bacterial species [82], except in members of the phyla *Cyanobacteria* and *Planctomyceota* [83]. A recent study identified that the coral tissue–associated *Endozoicomonas* spp. harbor a high count of WD40 repeats [84]. Proteins containing these repeat domains have been previously identified in sponges [85–87] and coral microbial symbionts [74, 80]. MAGs recovered in this study harbored a low abundance of WD40 and HEAT repeat proteins, suggesting that the coral skeletal microbiome might harbor distinct features from the coral tissue microbiome (Fig. 2A, B, Supplementary Data File). TPR proteins are also involved in mediating interactions between bacteria and eukaryotic hosts. TPR proteins were the most abundant group of ELPs in the MAGs, in congruence with earlier reports of TPR enrichment in bacteria compared to other ELPs [68], as well as bacteria cultured from the coral *Pocillopora damicornis* [88]. High counts of TRPs have been identified previously in the coral tissue microbiome members *Alteromonadales* and *Endozoicomonas* [80, 89]. In the present study, TPR proteins accounted for >80% of ELPs in 77 and 19 MAGs from *P. lutea* and *I. palifera*, respectively. Most of these MAGs belonged to *Alphaproteobacteria* and *Gammaproteobacteria* (Supplementary Data File). TPR-containing proteins are often involved in virulence-associated functions, such as translocation of virulence factors into the host [76], adhesion to the host, and blocking of phagolysosomal maturation [90, 91]. With high diversity of TPRs and other ELPs in the coral-associated bacteria, additional analysis is required to identify the mechanisms with which these bacteria interact with diverse microeukaryotes of the coral holobiont and the potential consequences of these interactions on the functioning of the holobiont.

### Roles of the skeletal microbiome in coral holobiont health and functioning

In addition to the reliance of corals on their symbiotic algae and bacterivory for carbon requirements [3, 7,92], recent studies have demonstrated the functional role of the coral microbiome in important metabolic pathways, including nitrogen, sulfur, and carbon metabolism [92]. We profiled the functional repertoire of MAGs recovered from the coral skeleton to gain more insights into the functional role of the skeletal microbiome in maintaining the health of the coral holobiont through stress removal and nutrient recycling.

MAGs belonging to diverse microbial lineages were identified to harbor genes for alleviating oxidative stress in the coral skeleton, with many MAGs harboring genes for DMSP synthesis and metabolisms (Fig. 1A). DMSP is an osmolyte, and its metabolic product DMS is a potent free radical scavenger and a climate-active gas [93]. Although coral microbiome members have been shown to metabolize DMSP and use it as the sole carbon source [9, 94–97], DMSP synthesis in the coral tissue microbiome has only recently been reported [98], indicating a substantial role of the tissue microbiome in coral sulfur cycling. The presence of DMSP synthesis genes in MAGs recovered from the coral skeleton of *P. lutea* and *I. palifera* provides further insights into the important role that the skeleton microbiome can play in alleviating oxidative stress and contributing to coral sulfur cycling. It is important to note that other DMSP-synthesizing bacteria could be present in the coral skeleton, potentially encoding the *dsy*B independent pathway [99]. Apart from DMSP synthesis and metabolism genes, an arsenal of other antioxidants, including SOD and Catalase genes, were also identified in the recovered MAGs from *P. lutea* and *I. palifera*, suggesting coral-associated bacteria harbor a diverse array of genes to mitigate oxidative stress (Supplementary Data File).

Micro-niches within the porous coral skeleton can harbor oxic pockets, predominantly within the green *Ostreobium*-dominated bands, whereas the bulk coral skeleton remains anoxic, facilitating anaerobic processes, including sulfate reduction [73,100, 101]. MAGs from *Desulfobacteria* and *Desulfarculia* harbored a complete dissimilatory sulfate reduction module in *P. lutea*, and MAGs from *Phycisphaerae* and *Planctomycetes* showed complete assimilatory sulfate reduction in both coral species along with members of different lineages harboring potential for sulfur metabolism (Fig. 3A, B). Assimilatory sulfate reduction was identified as the major pathway for sulfur metabolism in coral rubble [102]. Sulfate reducers, including *Desulfobacteria*, were first reported in the skeleton of *Goniastrea aspera* [101], but genes related to sulfur reduction were first identified in healthy and yellow bands of coral *Orbicella faveolata* [11]. Recently, metagenomic analysis of the skeleton of coral *I. palifera* and subsequent culturing and genomic analysis of dominant green sulfur bacteria (GSB) proposed a potential syntrophic relationship between GSB and SRB, where GSB can provide sulfate, which is used by SRB as an electron acceptor to generate biogenic H_2_S, which in turn is used by GSB as an electron donor [20, 103]. In this study, MAGs belonging to the genus *Chlorobium* (class: *Chlorobia*) and *Desulfobacter* (class: *Desulfobacteria*) were recovered from the skeleton of *P. lutea*, indicating the possibility of a similar syntrophic relationship in the skeleton (Fig. 3A, B, Supplementary Data File). Although no GSB MAGs were recovered from *I. palifera* in our study, this result was not surprising as an abundance of oxygenic phototrophs in the skeleton of *I. palifera* colonies from Heron Island has been previously reported [21]. The presence of MAGs from other microbial lineages, including phototrophic purple nonsulfur bacteria, with the potential to reduce sulfur and use H_2_S for energy production in MAGs recovered from both coral species, suggests complex interactions can exist between different members of the coral skeletal microbiome to develop syntrophic relationships. With skeletal architecture influencing the microbial community structure [22], whose metabolism influences the physiochemical gradients and microniches in the coral skeleton [73], a comprehensive spatial organization of the microbial community and heterogeneity of the biogeochemical activity is required for further insights into how different members of the coral skeletal microbiome interact.

Coral holobiont members are highly efficient in assimilating and retaining nitrogen, and the potential for it has been detected in many coral species, suggesting a key role of nitrogen cycling in holobiont functioning [104]. Coral reefs are net sources of fixed nitrogen [105], and cyanobacteria were earlier believed to be the main drivers of nitrogen fixation in corals [33, 34, 106]. Recent studies have revealed a ubiquitous presence of various nitrogen-fixing bacteria in corals [30–32], and diazotrophs may engage in important microbial and microbe–host interactions in the coral holobiont [107]. A previous genome-centric study found a low abundance of nitrogen-fixing genes in *P. lutea* [74, 97]. In contrast, we identified a diverse array of MAGs with the potential to fix nitrogen in both coral species, including MAGs from *Chlorobia* in *P. lutea* and *Cyanobacteria* in *I. palifera*. Ammonia, a product of nitrogen fixation, can be oxidized by ammonia-oxidizing bacteria and archaea. Archaea of the phylum *Theromoproteota* (*Crenarchaeota*,*Thaumarchaeota*) have been identified in different coral species and are capable of ammonia oxidation [74, 108]. We also found MAGs in the investigated coral species that belong to *Thermoproteota* and harbored *amoA* genes. These have also been identified in high cell densities in other corals species [15, 109], suggesting that archaea participate in nitrogen cycling in a range of corals.

Nitrogen can also be assimilated by microbes in the coral holobiont possessing nitrate reductases. We identified complete nitrogen assimilation and dissimilation modules in MAGs from different microbial lineages in both coral species (Fig. 3A, B). As the coral skeleton turns anoxic rapidly in darkness [100], denitrification and dissimilatory nitrate reduction (DNRA) activity have been hypothesized to be upregulated [15, 110]. With conditions, including near anoxia and limited nitrate availability in darkness, tailored for DNRA to outcompete denitrification, it was no surprise that only 3 MAGs recovered from *P. lutea* and *I. palifera* harbored a complete denitrification pathway. DNRA presents a significant nitrogen retention mechanism under dark conditions and can function as the principal pathway contributing to ammonia availability for assimilation in the coral [111].

## Conclusion

By applying genome-resolved metagenomics to the coral skeleton, we provide a comprehensive genomic view of the diversity and functional potential of the prokaryotic component of the skeletal microbiome. This study expands and enriches our understanding of the coral skeletal microbiome's role in holobiont functioning. Also, by undertaking a genome-centric study, we identified how the skeletal microbiome members harbor an arsenal of stress mediators, including DMSP synthesis and metabolism genes. These prokaryotic microbes have a diverse array of ELPs to establish symbiosis with the coral host and/or other eukaryotes in the coral holobiont. Importantly, we show that skeletal microbiomes from *P. lutea* and *I. palifera* have the potential to contribute to the nitrogen and sulfur cycling budget of the host. We provide a framework for future studies focused on identifying the key members of the skeletal holobiont and ascertaining their role in coral health, as well as how the skeletal microbiome functionally responds when the corals are under stress.

## Data Availability

All the sequencing data generated in this study are publicly available. MAGs assembled in this study are submitted to the NCBI genomes database under the bioproject PRJNA857095. Accession IDs of the MAGs are available in Supplementary Data File. All scripts, including R, bash, software parameters used, and supplementary data, are available on Figshare [52]. All supporting data and materials are also available in the *GigaScience* GigaDB database [112].

## Additional Files

**Supplementary Data File**. Dataset with details of all the MAGs recovered in this work, including genome statistics, genes of interest, and accession numbers.

**Supplementary Fig. S1**. Barplots depicting the metagenome bins recovered after each step. Only high-quality bins were used for performing all the analysis.

**Supplementary Fig. S2**. Boxplot for *P. lutea* and *I. palifera* MAGs' completeness and contamination stats.

**Supplementary Fig. S3**. Stacked barplots for relative abundance (>1%) of MAG taxonomic classes for *P. lutea* and *I. palifera*. p_***Aenigmatarchaeota***, p_***Nanoarchaeota***, and p_***Thermoproteota*** represent the archaea domain.

**Supplementary Fig. S4**. Heatmaps representing bacterial MAGs from (A) *P. lutea* and (B) I. *palifera* with at least 1 KEGG module >75% complete. MAGs are annotated at the class level.

giac127_GIGA-D-22-00206_Original_Submission

giac127_GIGA-D-22-00206_Revision_1

giac127_Response_to_Reviewer_Comments_Original_Submission

giac127_Reviewer_1_Report_Original_Submission Linda Wegley Kelly -- 10/3/2022 Reviewed

giac127_Reviewer_2_Report_Original_Submission Zewei Song -- 10/9/2022 Reviewed

giac127_Supplemental_Files

## Abbreviations

ARP: ankyrin repeat protein; DMSP: dimethylsulfoniopropionate; DNRA: dissimilatory nitrate reduction; ELP: eukaryotic-like protein; GSB: green sulfur bacteria; KEGG: Kyoto Encyclopedia of Genes and Genomes; MAG: metagenome-assembled genome; SOD: superoxide dismutase; SRB: sulfate-reducing bacteria; TPR: tetratricopeptide repeat.

## Authors' Contributions

K.T. and H.V. contributed to the conceptual development of the work and manuscript. F.R. and J.C. conducted the experiments. K.T. conducted the data analysis and wrote the first draft, addressed the reviewer comments/suggestions, and revised the manuscript. All authors contributed to the final edited version of the manuscript.

## Competing Interests

The authors declare that they have no competing interests.
