# Supplementary material for: Genomic view of the diversity and functional role of archaea and bacteria in the skeleton of the reef-building corals Porites lutea and Isopora palifera
Source: Gigascience. 2023 Jan 23;12:giac127. doi: 10.1093/gigascience/giac127 (PMC9868349; doi:10.1093/gigascience/giac127)
Supplement: giac127_GIGA-D-22-00206_Original_Submission [file giac127_giga-d-22-00206_original_submission.pdf]

GigaScience

Genomic view of the diversity and functional role of archaea and bacteria in the skeleton of the reef-building corals Porites lutea and Isopora palifera

--Manuscript Draft--

**Manuscript Number:**	GIGA-D-22-00206
**Full Title:**	Genomic view of the diversity and functional role of archaea and bacteria in the skeleton of the reef-building corals Porites lutea and Isopora palifera
**Article Type:**	Research
**Funding Information:**	Australian Research Council (DP200101613) — Prof. Mónica Medina<br>Prof. Michael Kühl<br>Prof. Linda L. Blackall<br>A/Prof. Heroen Verbruggen
**Abstract:**	At present our knowledge on the compartmentalization of coral holobiont microbiomes is highly skewed towards the millimetre-thin coral tissue, leaving the diverse coral skeleton microbiome underexplored. Here, we present a genome-centric view of the skeleton of the reef-building corals' Porites lutea and Isopora palifera , through a compendium of ~400 high-quality bacterial and archaeal metagenome-assembled genomes (MAGs), spanning 34 phyla and 57 microbial classes. Skeletal microbiomes harboured a diverse array of stress response genes, including dimethylsulfoniopropionate synthesis (dsy B) and metabolism (DMSP lyase). Furthermore, skeletal MAGs encoded an average of 22±15 genes in P. lutea and 28±23 in I. palifera with eukaryotic-like motifs thought to be involved in maintaining host association. We provide comprehensive insights into the putative functional role of the skeletal microbiome on key metabolic processes such as nitrogen fixation, dissimilatory and assimilatory nitrate, and sulphate reduction. Our study provides critical genomic resources for a better understanding of the coral skeletal microbiome and its role in holobiont functioning.
**Corresponding Author:**	Kshitij Tandon<br>University of Melbourne<br>VIC, VIC AUSTRALIA
**Corresponding Author Secondary Information:**	
**Corresponding Author's Institution:**	University of Melbourne
**Corresponding Author's Secondary Institution:**	
**First Author:**	Kshitij Tandon
**First Author Secondary Information:**	
**Order of Authors:**	Kshitij Tandon
	Francesco Ricci
	Joana Costa
	Mónica Medina
	Michael Kühl
	Linda L. Blackall
	Heroen Verbruggen
**Order of Authors Secondary Information:**	
**Additional Information:**	
**Question**	**Response**

Powered by Editorial Manager® and ProduXion Manager® from Aries Systems Corporation

Are you submitting this manuscript to a special series or article collection?	Yes
Please select an option from the menu: as follow-up to "Are you submitting this manuscript to a special series or article collection?"	Functional Metagenomics
**Experimental design and statistics**<br><br>Full details of the experimental design and statistical methods used should be given in the Methods section, as detailed in our Minimum Standards Reporting Checklist. Information essential to interpreting the data presented should be made available in the figure legends.<br><br>Have you included all the information requested in your manuscript?	Yes
**Resources**<br><br>A description of all resources used, including antibodies, cell lines, animals and software tools, with enough information to allow them to be uniquely identified, should be included in the Methods section. Authors are strongly encouraged to cite Research Resource Identifiers (RRIDs) for antibodies, model organisms and tools, where possible.<br><br>Have you included the information requested as detailed in our Minimum Standards Reporting Checklist?	Yes
**Availability of data and materials**<br><br>All datasets and code on which the conclusions of the paper rely must be either included in your submission or deposited in publicly available repositories (where available and ethically appropriate), referencing such data using a unique identifier in the references and in the “Availability of Data and Materials”	Yes

section of your manuscript.<br><br>Have you have met the above requirement as detailed in our Minimum Standards Reporting Checklist?	

Manuscript File Click here to access/download;Manuscript;Tandon K et al .docx

Genomic view of the diversity and functional role of archaea and bacteria in the skeleton of the reef-building corals *Porites lutea* and *Isopora palifera*

Kshitij Tandon[1#], Francesco Ricci[1,2], Joana Costa[1], Mónica Medina[3], Michael Kühl[4], Linda L. Blackall[1], Heroen Verbruggen[1]

[1] School of BioSciences, University of Melbourne, Parkville 3010, Australia

[2] Biological, Earth and Environmental Sciences, The University of New South Wales, Kensington, NSW 2052, Australia

[3] Department of Biology, Pennsylvania State University, University Park, PA 16802, USA

[4] Marine Biological Section, Department of Biology, University of Copenhagen, Strandpromenaden 5, DK-3000 Helsingør, Denmark

[#] Corresponding author: Kshitij Tandon

Corresponding author email: k.tandon@unimelb.edu.au

Abstract

At present our knowledge on the compartmentalization of coral holobiont microbiomes is highly skewed towards the millimetre-thin coral tissue, leaving the diverse coral skeleton microbiome underexplored. Here, we present a genome-centric view of the skeleton of the reef-building corals' *Porites lutea* and *Isopora palifera*, through a compendium of ~400 high-quality bacterial and archaeal metagenome-assembled genomes (MAGs), spanning 34 phyla and 57 microbial classes. Skeletal microbiomes harboured a diverse array of stress response

genes, including dimethylsulfoniopropionate synthesis (*dsy*B) and metabolism (DMSP lyase). Furthermore, skeletal MAGs encoded an average of 22±15 genes in *P. lutea* and 28±23 in *I. palifera* with eukaryotic-like motifs thought to be involved in maintaining host association. We provide comprehensive insights into the putative functional role of the skeletal microbiome on key metabolic processes such as nitrogen fixation, dissimilatory and assimilatory nitrate, and sulphate reduction. Our study provides critical genomic resources for a better understanding of the coral skeletal microbiome and its role in holobiont functioning.

Introduction

Symbiont-bearing, reef-building corals harbour diverse microbiomes forming a multi-species consortium termed the coral holobiont [1]. Much like other multicellular organisms, corals rely on their microbiome for health and functioning [2,3]. A rapid decline in coral reefs across the globe has shifted the focus to characterising the functional role of coral-associated bacteria, unarguably the most diverse members of the coral holobiont. Bacteria have the potential for utilisation in developing effective assisted evolution strategies such as coral probiotics [4,5] and microbiome manipulation [6] to protect coral reefs. Recent studies showed that coral-associated bacteria play significant roles (e.g., in nutrient recycling[7–9] and protection against pathogens [7] that can govern coral health). Bacterial community composition profiles also serve as indicators of coral health exhibiting shifts to less diverse microbiomes with a stronger predominance of pathogens during dysbiosis [10–12]. However, it is important to note that most of our present knowledge on coral microbiomes and their role in coral holobiont fitness and health stems from investigations of the millimetre thick coral tissue layer. This layer is spread over a voluminous calcium carbonate structure (i.e., the coral skeleton), which harbours endolithic microorganisms. This microhabitat is often neglected in coral microbiome research but represents a key ecological niche for microorganisms in the coral holobiont [13].

The bulk of the coral skeleton, except the tissue-skeleton interface and upper millimetres of the skeleton in shallow-water corals, receives low irradiance and exhibits a broader array of microenvironmental dynamics than the coral tissue [3]. Metabarcoding surveys have revealed vast microbial and microeukaryotic diversity, including archaea [14,15], bacteria [16–21], fungi [22–24], and protists such as endolithic green algae in the genus *Ostreobium* [16],

showing that the skeletal microbiome differs significantly from that of other coral compartments [25]. Microboring, filamentous green algae (*Ostreobium* spp.) form conspicuous green bands in the skeletons of several coral species. *Ostreobium* can play an active role in both providing carbon substrates to coral hosts during thermal stress-induced bleaching and facilitating coral recovery [26–28]. Endolithic microbes have also been reported to actively participate in nutrient recycling and primary productivity [13,29]. Functional characterisation of complex microbial assemblages in the coral skeleton has mainly relied on selective amplification of target genes, (e.g., *nifH* [30–32]), experimental approaches such as the acetylene reduction technique, and isotope labelling for probing N_2 fixation and other metabolic activities[33,34]. While such studies have yielded important insights into the coral skeleton niche, a genome-centric view of the coral skeletal microbiome and its functional potential remains elusive.

Whole-genome shotgun sequencing complemented with metagenome binning has been applied to recover genomes of dominant green-sulphur bacteria belonging to the genus *Prosthecochloris* in the skeleton of coral *Isopora palifera* [19,35]. These studies are the only shallow-depth genome-centric research conducted to date on the coral skeleton, with a combination of FISH-nanoSIMS and the acetylene reduction assay to confirm the dinitrogen-fixing ability of dominant anaerobic phototrophs. A recent study used a combination of metabarcoding, gene- and genome-centric metagenomics to shed light on the role of endolithic microbiome in coral bleaching susceptibility [36]. The limited insights into the broader functional potential of coral skeletal microbiomes hamper our ability to identify key roles of the skeleton microbiota within the coral holobiont.

To address these knowledge gaps, we applied a deep sequencing metagenomics approach to obtain metagenome-assembled genomes (MAGs) from bacteria and archaea residing in the skeletons of the two reef-building corals *Porites lutea* and *Isopora palifera*. We further explored the potential of these microbiome members to provide essential functions to the coral holobiont in terms of engaging in symbiosis with the host, their ability to mitigate oxidative stress, and their role in biogeochemical nutrient cycling.

Materials and Methods

Sample collection and processing

Fragments from five individual healthy-looking colonies of *Porites lutea* and *Isopora palifera* were each collected at low tide (<1 m depth) from the research zone of the Heron Island reef flat, central Great Barrier Reef (23°44'S, 151°91'E), in January 2020. The fragments were collected using a sterile hammer and chisel and were immediately placed in sterile zip-lock polyethylene bags in seawater. Coral tissue was removed from the fragments using a Waterpik and sterile seawater (SSW). Coral fragments with only skeletons were snap-frozen by immersion in liquid nitrogen and stored at -80°C until processing.

DNA isolation, library preparation and Whole Metagenome Sequencing

Total DNA was extracted using DNeasy PowerSoil Pro Kit (Qiagen) as per the manufacturer's protocol. Extracted DNA samples were sent to BGI Tech Solutions (Hong Kong) for library preparation and sequencing on individual lanes per sample using DNBSeq (2x150). On average, we obtained > 327 million and >298 million read pairs for *P. lutea* and *I. palifera* respectively.

Read QC, Trimming and removal of host-related reads

Paired-end reads were quality checked using FASTQC (https://www.bioinformatics.babraham.ac.uk/projects/fastqc/) and multiQC [37]. Reads were trimmed with trimmomatic v0.38 [38] with the following parameters *HEADCROP:5 SLIDINGWINDOW:4:20 MINLEN:30*. Trimmed reads from five *P. lutea* samples were mapped to its draft genome downloaded from (https://plut.reefgenomics.org/download/), using bowtie2 with default settings[39]. Unmapped paired-end reads were extracted using samtools v1.7[40] . Two samples, PL23b_i and PL25b_i had 48.19% and 63.66% reads mapping to the *P. lutea* genome, and an additional full lane of sequencing was performed for them and processed with the same specifications. Only paired-end unmapped reads were used for *de novo* metagenome assembly. Paired end trimmed reads from *I. palifera* metagenome samples were directly assembled as the host genome is not available.

Metagenome assembly, Binning and dereplication

Metagenome assembly was performed on individual samples using MegaHIT v1.2.9[41] with kmers 33,55,77,99 and a minimum contig length of 1000. Resultant contigs per sample were binned using Concoct v1.0.0[42], Maxbin2 v2.2.6[43] and Metabat2 v2.12.1 [44] as implemented in MetaWrap v1.3.2 [45]. One sample of *I. palifera* (IP31a_i) yielded no bins from Maxbin2 and this sample was additionally binned using Metabat1 [46]. Obtained bins were then refined using the bin_refinement module of MetaWrap with parameters *completeness >= 50%* and *contamination <=10%*. Refined bins from all the samples were pooled and dereplicated using dRep v3.0.0 [47] using default parameters. CheckM v1.0.12

[48] was used to estimate the completeness and contamination statistics of dereplicated bins; only bins with at least 80% completeness and less than 10% contamination were selected for downstream analysis. Bins were subjected to CAT and BAT v5.2.3 [49] to identify misbinned contigs based on taxonomic affiliation in a bin using default parameters. Contigs annotated as Eukaryota were removed from the bins using a custom python script (https://doi.org/10.26188/20364108.v1). Completeness and contamination statistics were again evaluated as above. We categorised the bins into high-quality (completeness >80% and contamination <10%) and medium-low quality (completeness >50-<80% and contamination <10%) based on CheckM completeness and contamination statistics. Only high-quality bins were used for downstream processing and were called Metagenome-assembled-genomes (MAGs).

Taxonomic assignment and relative coverage of MAGs

Taxonomic assignment of each dereplicated and CAT and BAT corrected bin was performed based on the Genome Taxonomy Database release202 using the *classify_wf* approach implemented in GTDB-Tk [50]. GTDB-Tk classifies MAGs by placing them in a referenced tree inferred using a set of 120 bacterial and 122 archaeal concatenated gene markers using a combination of FastANI [51] and pplacer [52]. We mapped each coral species-specific metagenomic trimmed paired-read set to coral-species-specific MAGs using BBMap [53], which generates coverage information using *pileup*. To calculate the relative coverage as a proxy for abundance across the samples, we calculated the average coverage per contig per MAGs and converted it to a relative coverage profile to represent the genome coverage per sample. Stacked bar-plots were generated in R v4.0.2 [54] using ggplot2 [55] to represent relative-read coverage of MAGs per coral colony.

Phylogenetic tree building and visualisation

Archaeal and bacterial phylogenetic trees were constructed by providing respective concatenated marker gene alignments generated by GTDB-Tk to IQ-TREE v1.6.1 [56] with LG+G selected as the model and 1000 ultrafast bootstraps. Phylogenetic trees with genome statistics across microbial lineages and distribution of genes-of-interest (see details below) were visualised using the iTOL v6 [57].

Gene prediction, annotation and metabolic potential

Prodigal v2.6.3 [58] implemented in Prokka v1.14.5 [59] was used for gene prediction. Predicted genes per MAGs were then provided to Interproscan v5.53.87 [60] to search for protein family (Pfam) ids (*-appl Pfam*), with -evalue cutoff of -1e-5. Unique hits from filtered output were searched for genes-of-interest, including Eukaryotic like proteins (ELPs): WD40 repeats proteins (WD40) (PF00400 and PF07676), Ankyrin repeat proteins (ARP) (PF00023 and PF13857), HEAT repeat proteins (HEAT) (PF13646), Tetratricopeptide repeat (TPR) (PF00515, PF07719, PF09976, PF13174, PF13181, PF13371, PF13374, PF13424, PF13428, PF13429, PF13431, PF13432, PF14559, PF14561 and PF16918), Nitrogen fixation (*nif*H) (PF00142), Dimethylsulfoniopropionate metabolism (DMSP) synthesis (*dsy*B) (PF00891 and PF16864) and catabolism (DMSP_lyase) (PF16867), superoxide dismutase (SOD) (PF00080, PF00081 and PF02777), catalase (PF00199) and Ammonia oxidation (AmoA) (PF12942). METABOLIC-G, implemented in METABOLIC [61] was used for annotation of Kyoto Encyclopaedia of Genes and Genomes (KEGG) pathways to determine the functional potential of MAGs using the

following parameters *-m-cutoff 0.50*. Results from METABOLIC-G on a per MAG level were parsed and collated using CSV/TSV tool kit (https://bioinf.shenwei.me/csvtk/). Collated output was combined as a matrix and used as input to EnrichM (https://github.com/geronimp/enrichM) *classify* workflow for calculating the completeness of predicted KEGG modules. KEGG modules with >75% completeness for nitrogen metabolism, sulphur metabolism and anoxygenic photosynthesis in any samples were plotted as a heatmap using *pheatmap* [62] in R.

Data Availability

All the sequencing data generated in this study is publicly available. MAGs assembled in this study are submitted to the NCBI genomes database under the bioproject PRJNA857095. Accession IDs of the MAGs are available in Supplementary data file. All scripts, including R, bash, software parameters used and supplementary data are available on figshare https://doi.org/10.26188/20364108.v1.

Results

Sequencing overview, P. lutea and *I. palifera* skeletal microbiome

We sequenced a total of 1.6383 billion read pairs for *P. lutea* (3.39%-63.66% host) and 1.2952 billion read pairs for *I. palifera* samples (Supplementary data file). We obtained 250 high-quality MAGs from *P. lutea* (average completeness (± standard deviation): 92.89±5.53% and contamination: 2.22±1.72%) and 143 from *I. palifera* (avg. completeness: 93.89±5.46% and contamination: 1.91±1.61%) (Supplementary Figures S1 and S2). Of the 250 *P. lutea* MAGs, 235 were bacterial and 15 archaeal and 141 of *I. palifera* 143 MAGs were bacterial and only 2

archaeal, based on GTDB-tk classification (Figure 1 a and b). A total of 113 MAGs (69 *P. lutea* and 44 *I. palifera*) had at least one copy of the 16S rRNA gene predicted in them (Figure 1 a and b; Supplementary data file).

These MAGs spanned the vast majority of microbial lineages (34 phyla and 57 classes) in the coral skeleton (Figure 1**,** Supplementary data file), including bacteria from phyla *Proteobacteria* (147 MAGs), *Bacteroidota* (75), *Planctomycetota* (42), *Desulfobacterota* (12, including lineages B and F), *Firmicutes* (12, including lineages A, F, G and H), *Cyanobacteria* (11), *Verrucomicrobiota* (11), *Chloroflexota* (10), *Myxococcota* (9), *Gemmatimonadota* (5), *Bdellovibrionota* (5), *Actinobacteriota* (4), *Chlamydiota* (4), *Patescibacteria* (4), SAR324 (4), *Acidobacteriota* (3), *Spirochaetota* (3), *Bipolaricaulota* (2), *Calditrichota* (2), AABM5-125-24 (1), DSWW01 (1), *Elusimicrobiota* (1), *Fibrobacterota* (1), *Marinisomatota* (1), *Nitrospinota* (1), *Omnitrophota* (1), SM23-31 (1), *Sumerlaeota* (1), *Zixibacteria* (1) and archeal phyla *Nanoarchaeota* (7), *Thermoplasmatota* (4), *Thermoproteota* (3) *Aenigmatarchaeota* (2, including lineage A) and *Iainarchaeota* (1).

P. lutea **and** ***I. palifera*** **harbour different skeletal microbiome**

Comparing microbial communities recovered from MAGs which meet completeness (≥90% and contamination (≤10%) thresholds, and basing our results on the presence and absence of MAGs from the two coral species, we identified some MAGs (*Actinobacteriota, Calditrichota, Sumerlaeota* and *Zixibacter*) to be unique to *I. palifera*, and some other MAGs (AABM5-125-24, *Bipolaricaulota, Desulfobacterota, DSWW01, Elusimicrobiota, Fibrobacterota, Firmicutes, Marinisomatota, Nitrospinota, Omnitrophota, Patesibacteria,* SAR324 and SM23-31) to be

unique to *P. lutea*. We recovered one archeal MAG each of *Thermoproteota* and *Nanoarchaeota* from *I. palifera* metagenomes, whereas *Iainarchaeota*, *Aenigmatarchaeota* and *Thermoplasmatota* MAGs were recovered from *P. lutea* metagenomes.

MAGs recovered from *P. lutea* were differentially abundant among colonies, whereas the relative abundance of MAGs appeared stable among the colonies of *I. palifera* (Supplementary Figure S3). *P. lutea* skeletal samples were dominated by MAGs from bacterial classes *Alphaproteobacteria, Vampirovibrionia* and *Planctomycetes* and one sample (PL25b) was also dominated by archaeal phyla *Thermoproteota* (Supplementary Figure S3). In contrast, *I. palifera* skeletal samples were dominated by MAGs from bacterial classes *Bacteroidia, Cyanobacteria, Anaerolineae* and *Polygania*, with one colony (IP29b) harbouring a relatively high abundance (45.76%) of *Cyanobacteria* MAG (IP29b_bin.176) (Supplementary Figure S3).

Skeletal bacteria show the potential to engage in symbiosis with eukaryotes

Recovered MAGs on an average encoded 0.56±0.31% (*P. lutea*), 0.61±0.38 % (*I. palifera*) Eukaryotic Like Proteins (ELPs) per genome. MAGs had a broader range of ELPs including, WD40 (*P. lutea*: 2.65±4.06; *I. palifera*:4.68±6.32) and HEAT repeats (*P. lutea*: 2.65±5.04; *I. palifera*: 3.28±4.77) (Figure 2 a and b). The most abundant group of ELP in MAGs from *P. lutea* and *I. palifera* were TPRs (*P. lutea:* TPR_16, Pfam: PF13432, avg. proteins: 3.88±3.75; *I. palifera:* TPR_12: Pfam: PF13424, avg. proteins:4.94±8.27). MAGs harboured relatively low numbers of WD40 and HEAT repeat proteins, with the highest count in a MAG from Candidate phylum SM23-31 (37 WD40 repeat proteins), in *P. lutea* (PL23a_bin.125) and a MAG from class *Bacteroidia* (39 WD40 repeat proteins) in *I. palifera* (IP29b_bin.15). A MAG belonging to class UBA1135 (phylum: *Planctomycetes*) harboured 47 and 25 HEAT repeat proteins in *P.*

lutea (PL25a_bin.29) and *I. palifera* (IP29b_bin.26, respectively (Supplementary data file). ARPs were the least abundant ELPs in the MAGs (*P. lutea*: 2.09±2.6; *I. palifera*: 2.58±2.25). Out of 235 bacterial MAGs from *P. lutea*, no ARPs were identified in 72 MAGs, 63 MAGs had only 1 ARP and there were 9 MAGs encoding more than 10 copies of ARP. In contrast, out of 141 bacterial MAGs from *I. palifera*, 23 had none, 36 MAGs had only 1 copy and 2 MAGs had 10 ARPs (Supplementary data file). Microbes are considered host-associated if they devote more than 0.2% of their total gene repertoire to ARPs [63]. Keeping this conservative threshold as identified earlier, we identified only 10 MAGs belonging to 6 bacterial phyla from *P. lutea* and 5 MAGs from 3 phyla from *I. palifera*, meeting this criterion (Figure 2 a and b). Further, all 3 *Chlamydia* MAGs from *P. lutea* and 2 *Bdellovibrionia* MAGs from *I. palifera* encoded >0.2% ARPs.

The skeletal microbiome harbours an array of oxidative stress alleviators

Approximately half of the *P. lutea* bacterial MAGs (114) had at least one copy of the *dsyB* (PF00891, PF16864) gene, conferring the ability to synthesise DMSP and 13 MAGs had at least one copy of DMSP_lyase (PF16867) gene able to metabolise DMSP to other potent antioxidants (Figure 1a). Though the ability to synthesise DMSP was identified in 48.5% of MAGs, only 8 MAGs have at least one copy of both *dsy*B and DMSP_lyase genes (Figure 1a), with 7 of these belonging to the class *Alphaproteobacteria* and 1 to *Gammaproteobacteria* (Supplementary data file). The catalase gene (PF00199) was identified in 13 MAGs. At least one copy of the superoxide dismutase, *SOD* gene (including SODC (PF00080) and *SOD_Fe_N* (PF00081)) was identified in 94 bacterial MAGs. In contrast, out of 141 bacterial *I. palifera* MAGs, 58 had at least one copy of the *dsy*B gene and 16 MAGs had a copy of *DMSP_lyase* (Figure 1a). Further, only 13 MAGs belonging to class *Alphaproteobacteria* (11 MAGs),

Anaerolineae (1 MAGs) and *Acidimicrobia* had at least a copy of *dsyB* and *DMSP_lyase* genes (Supplementary data file). SOD genes were annotated in 74 MAGs and catalase genes were identified in 5 MAGs only.

Skeletal archaea and bacteria engage in nitrogen and sulphur metabolism

We identified that 87 *P. lutea* and 45 *I. palifera* MAGs harbour the potential to fix nitrogen with at least one copy of the *nif*H gene (PF00142) (Figure 1a). Ammonia oxidation, *Amo*A gene, was identified in 2 and 1 Archeal MAGs from *P. lutea* and *I. palifera*, respectively (Figure 1b). We analysed the processes involved in nitrogen cycling, including nitrification, denitrification, nitrogen fixation, and assimilatory and dissimilatory nitrate reduction to obtain comprehensive insights and understanding of nitrogen metabolism by the members of the coral skeleton microbiome. The nitrogen fixation module (M00175) was identified as complete in several MAGs, with 10 MAGs of *Chlorobia* and 3 of *Clostridia* encoding complete nitrogen fixation modules in *P. lutea* (Figure 3a) and 2 *Cyanobacteria* MAGs, 4 *Alphaproteobacteria* MAGs and 1 *Planctomycetes* MAG harbouring the potential to fix nitrogen in *I. palifera* (Figure 3a). Interestingly, the oxygen-dependent regulatory nitrogen fixation module (M00524) mediated by FixL-FixJ genes was also complete in several MAGs belonging to *Alphaproteobacteria, Gammaproteobacteria, Phycisphaerae* and *Planctomycetes* (Figure 3b). The dissimilatory nitrate reduction module (M00530), producing ammonia from nitrate was complete in MAGs spanning different bacterial classes in the two coral species (Figure 3a and 3b). However, assimilatory nitrate reduction (M00531) ability was poorly represented, with only MAGs from *Cyanobacteria* and *Alphaproteobacteria* encoding the complete module. The denitrification module was complete in 1 MAG each belonging to

Gammaproteobacteria in *P. lutea* (Figure 3a) and *Alphaproteobacteria*, Anaerolineae and *Bacteroidia* in *I. palifera* (Figure 3b).

In oceans, sulphur is available as inorganic sulphate that can be assimilated by microbes into organic compounds. We searched for the ability of coral skeletal microbes to assimilate inorganic sulphur and use it to produce organic compounds as well as for energy-yielding purposes. We identified MAGs of sulphate reducing bacteria (SRB), including members of *Desulfobacteria*, *Desulfarculia* and SAR324 encoding the complete dissimilatory sulphate reduction module (M00596), along with a few MAGs belonging to Gammaproteobacteria, *Chlorobia* and *Alphaproteobacteria* in *P. lutea* (Figure 3a), whereas in *I. palifera* only 2 *Gammaproteobacteria* MAGs had the complete module (Figure 3b). We found complete assimilatory sulphate reduction modules in MAGs from several lineages, including *Alphaproteobacteria, Bacterodia, Binatia, Gammaproteobacteria, Phycisphaerae, Planktomycetes* and *Verrucomicrobiae* from the two coral species (Figure 3a and 3b). Further, complete Anoxygenic photosystem II module (M00597) was identified in several MAGs belonging to purple sulphur and purple non-sulphur bacteria from different classes, including *Alphaproteobacteria, Anaerolineae, Gammaproteobacteria, Gemmatimonadetes* and others in both coral species (Figure 3a and 3b). Bacteria harbouring this module have the potential to use H_2S produced by assimilatory and dissimilatory reduction of sulphate as primary electron donor.

Discussion

With coral reefs under significant pressure across the globe due to climate change and other stressors derived from anthropogenic activities, coral microbiome research has recently been

gaining a lot of traction for the development of coral probiotic and assisted evolution strategies, including microbiome manipulation and buildout of thermotolerant microbial symbionts to protect reefs [6,8,64–67]. Here, we describe a compendium of bacterial and archaeal high-quality MAGs recovered from skeletons of two dominant reef-forming coral species, *P. lutea* and *I. palifera*. Our results provide an unprecedented view of the coral skeletal microbiome, permitting more detailed discussion of the community composition, the ability of endolithic microbes to form symbiosis with eukaryotes within the coral skeleton and the functional roles these endoliths can play in nutrient cycling and holobiont functioning.

Genome-centric view of the skeletal microbiome

The biggest challenge in working with host microbiomes is the contamination from the host DNA, which is often compounded by the lack of host genome required to remove host related sequencing reads. This was true in our study, with *P. lutea* skeletal samples showing varying proportions of host reads (Supplementary Table S1) and lack of *I. palifera* genome to account for host related reads in the samples. Considering there was some coral tissue sequenced, it is reasonable to assume that some of the MAGs reported in this study might also not be exclusively found in the coral skeleton. In that context, it is important to note that there is no strict boundary between the coral tissue and the skeleton and upper layers of the coral skeleton also harbour coral tissue-associated bacteria as reported in our earlier study [21]. Using deep metagenomic sequencing, we recovered a compendium of 435 bacterial and 15 archaeal high-quality MAGs from the skeleton of *P. lutea* and *I. palifera* corals (Figure 1a and b). The community composition of recovered MAGs reflects on studies using marker gene surveys to profile the coral skeletal microbial community often dominated by members of class *Alphaproteobacteria, Clostridia* and *Chlorobia* for *P. lutea* and *Bacteroidia, Anaerolineae*

(Phylum: *Chloroflexota*) and *Chlorobia* for *I. palifera* [16–18,20,21,68]. The community composition of MAGs recovered from *P. lutea* skeletons studied here was vastly different from 52 MAGs reported from *P. lutea* tissue in a recent study [69]. MAGs recovered from *P. lutea* tissue belonged to *Poribacteria, Actinobacteriota*, *Dadabacteria*, *Latescibacterota*, UBP10, which were not recovered in our study. But we did recover MAGs belonging to the archaeal class *Nitrososphaeria* and a few bacterial classes. Further, we identified a similar MAG community composition although with significantly more diversity of MAGs recovered in our study compared to a recent study using genome-centric approach on coral skeleton [36].In light of these comparisons, we provide an exhaustive collection of skeletal dominated coral-associated bacterial and archaeal MAGs.

Skeletal microbiomes harbour an array of ELPs to form stable symbiosis with eukaryotes in the coral holobiont

The coral holobiont is highly complex with the presence of several microeukaryotes and a high microbial diversity. Corals and potentially these microeukaryotes rely on prokaryotic microbes for fulfilling their metabolic requirements. Therefore, these microbes must harbour the genetic machinery to interact with the host without eliciting the host's immune response, (e.g., by harbouring proteins containing eukaryotic-like repeats or ELPs). Coral-associated bacteria harbouring these ELPs also have the potential to interact with other microeukaryotes present in the coral skeleton, including endolithic microalgae (e.g., *Ostreobium, Phaeophila* [17], sponges and corallimorphs), endolithic fungi including *Ascomycota* and *Basidiomycota* [20,22,70] among others. Proteins containing these repeats are commonly associated with additional functional domains such as lipid metabolism and mediating ubiquitination,

therefore these are likely to engage host proteins directly [71]. Although ELPs have been prevalent in bacteria associated with marine invertebrates, including corals, the presence of different types of ELPs in bacterial genomes raises the question of their diverse roles and how one should weigh the importance of one type of ELP over others. A recent comprehensive study suggests that ELPs abundance is determined by different factors [63]. ARP abundance is more related to the lifestyle of the bacteria, whereas TPR abundance is determined by phylogenetic history rather than lifestyle [63,72].

Ankyrin repeats, which span 30-40 amino acids and exclusively function in mediating protein-protein interactions [73], are a well-characterised group of ELPs. In *Escherichia coli*, Ankyrin repeat-containing genes, when expressed, were shown to help modulate phagocytosis by sponge amoebocytes, suggesting a possible mechanism by which symbionts can evade digestion from host cells and establish symbiosis [74]. Coral-associated bacteria have been reported to contain a wide array of ARPs, with high gene copies in tissue-associated bacteria, including *Endozoicomonas* [9], *Poribacteria* [69], and low dominance in *Vibrio* strains [75]. In the present study, members of diverse microbial lineages were identified to harbour ARPs ranging from 1-13 proteins in *P. lutea*-associated bacteria and 1-10 proteins in *I. palifera*-associated bacterial MAGs. Only a handful of MAGs devoted more than 0.2% of total proteins to ARPs in the current study, suggesting that only a few microbial symbionts that colonise the coral skeleton potentially have a strictly host-associated lifestyle and could be obligate symbionts. *Chlamydiae* are strictly intracellular and therefore intimately reliant on their hosts [76], three high-quality MAGs recovered from *P. lutea* colonies devoted more than 0.2% of their proteome to ARPs (Figure 2a).

WD40 proteins are widespread in eukaryotes but are rare in bacterial species[77], except in members of the phylum *Cyanobacteria* and *Planctomyceota* [78]. A recent study identified the coral tissue associated *Endozoicomonas* spp. harbour high count of WD40 repeats [79]. Proteins containing these repeat domains have been previously identified in sponges[80–82] and coral microbial symbionts [69,75]. MAGs recovered in this study harboured a low abundance of WD40 and HEAT repeat proteins, suggesting that the coral skeletal microbiome might harbour distinct features from the coral-tissue microbiome (Figure 2a and 2b, Supplementary data file). TPR proteins are also involved in mediating interactions between bacteria and eukaryotic hosts. TPR proteins were the most abundant group of ELPs in the MAGs, in congruence with earlier reports of TPR enrichment in bacteria compared to other ELPs [63] as well as bacteria cultured from the coral *Pocillopora damicornis*[83]. High counts of TRPs have been identified previously in the coral tissue microbiome members *Alteromonadales* and *Endozoicomonas* [75,84]. In the present study, TPR proteins accounted for >80% of ELPs in 77 and 19 MAGs from *P. lutea* and *I. palifera*, respectively. Most of these MAGs belonged to *Alphaproteobacteria* and *Gammaproteobacteria* (Supplementary data file). TPR-containing proteins are often involved in virulence associated functions, such as translocation of virulence factors into the host [71], adhesion to the host and blocking of phagolysosomal maturation [85,86]. With high-diversity of TPRs and other ELPs in the coral-associated bacteria, additional analysis is required to identify the mechanisms with which these bacteria interact with diverse microeukaryotes of the coral holobiont and the potential consequences of these interactions on the functioning of the holobiont.

Roles of the skeletal microbiome in coral holobiont health and functioning

In addition to the reliance of corals on their symbiotic algae and bacterivory for carbon requirements [3,7,87], recent studies have demonstrated the functional role of the coral microbiome in important metabolic pathways, including nitrogen, sulphur and carbon metabolism [87]. We profiled the functional repertoire of MAGs recovered from the coral skeleton to gain more insights into the functional role of the skeletal microbiome in maintaining the health of coral holobiont through stress removal and nutrient recycling.

MAGs belonging to diverse microbial lineages were identified to harbour genes for alleviating oxidative stress in the coral skeleton, with many MAGs harbouring genes for DMSP synthesis and metabolisms (Figure 1a). DMSP is an osmolyte and its metabolic product DMS is a potent free radical scavenger and a climate-active gas [88]. Although coral microbiome members have been shown to metabolise DMSP and use it as the sole carbon source [9,89–92], DMSP synthesis in the coral tissue microbiome has only recently been reported [93], indicating a substantial role of tissue microbiome in coral sulphur cycling. The presence of DMSP synthesis genes in MAGs recovered from the coral skeleton of *P. lutea* and *I. palifera* provides further insights into the important role that the skeleton microbiome can play in alleviating oxidative stress and contributing to coral sulphur cycling. It is important to note that other DMSP-synthesising bacteria could be present in the coral skeleton, potentially encoding the *dsyB* independent pathway [94]. Apart from DMSP synthesis and metabolism genes, an arsenal of other antioxidants, including SOD and Catalase genes were also identified in the recovered MAGs from *P. lutea* and *I. palifera* suggesting coral-associated bacteria harbour a diverse array of genes to mitigate oxidative stress. (Supplementary data file).

Micro-niches within the porous coral skeleton can harbour oxic pockets, predominantly within the green *Ostreobium*-dominated bands, whereas the bulk coral skeleton remains anoxic, facilitating anaerobic processes, including sulphate reduction [68,95,96]. MAGs from *Desulfobacteria* and *Desulfarculia* harboured complete dissimilatory sulphate reduction module in *P. lutea* and MAGs from *Phycisphaerae* and *Planctomycetes* showed complete assimilatory sulphate reduction in both coral species along with members of different lineages harbouring potential for sulphur metabolism (Figure 3a and b). Assimilatory sulphate reduction was identified as the major pathway for sulphur metabolism in coral rubble[97]. Sulphate reducers, including *Desulfobacteria,* were first reported in the skeleton of *Goniastrea aspera* [96], but genes related to sulphur reduction were first identified in healthy and yellow bands of coral *Orbicella faveolata* [11]. Recently, metagenomic analysis of the skeleton of coral *Isopora palifera* and subsequent culturing and genomic analysis of dominant green sulphur bacteria (GSB) proposed a potential syntrophic relationship between GSB and SRB, where GSB can provide sulphate, which is used by SRB as an electron acceptor to generate biogenic H_2S, which in turn is used by GSB as electron donor [19,98]. In this study MAGs belonging to the genus *Chlorobium* (class: *Chlorobia)* and *Desulfobacter* (Class: *Desulfobacteria*) were recovered from the skeleton of *P. lutea* indicating the possibility of a similar syntrophic relationship in the skeleton (Figure 3a and b; Supplementary data file). Although no GSB MAGs were recovered from *I. palifera* in our study, this result was not surprising as an abundance of oxygenic phototrophs in the skeleton of *I. palifera* colonies from Heron Island has been previously reported [20]. The presence of MAGs from other microbial lineages, including phototrophic purple non-sulphur bacteria, with the potential to reduce sulphur and use H_2S for energy production in MAGs recovered from both coral species, suggests complex interactions can exist between different members of the coral skeletal

microbiome to develop syntrophic relationships. With skeletal architecture influencing the microbial community structure [21], whose metabolism influences the physiochemical gradients and microniches in the coral skeleton [68], a comprehensive spatial organisation of the microbial community and heterogeneity of the biogeochemical activity is required for further insights into how different members of the coral skeletal microbiome interact.

Coral holobiont members are highly efficient in assimilating and retaining nitrogen and the potential for it has been detected in many coral species, suggesting a key role of nitrogen cycling in holobiont functioning [99]. Coral reefs are net sinks of fixed nitrogen [100] and cyanobacteria were earlier believed to be the main drivers of nitrogen fixation in corals [33,34,101]. Recent studies have revealed a ubiquitous presence of various nitrogen-fixing bacteria in corals [30–32], and diazotrophs may engage in important microbial and microbe-host interactions in the coral holobiont[102]. A previous genome-centric study found a low abundance of nitrogen-fixing genes in *P. lutea* [69,92]. In contrast, we identified a diverse array of MAGs with the potential to fix nitrogen in both coral species, including MAGs from *Chlorobia* in *P. lutea* and *Cyanobacteria* in *I. palifera*. Ammonia, a product of nitrogen fixation, can be oxidised by ammonia oxidising Bacteria and Archaea. Archaea of the phylum *Theromoproteota* (*Crenarchaeota, Thaumarchaeota*) have been identified in different coral species and are capable of ammonia oxidation [69,103]. We also found MAGs in the investigated coral species that belong to *Thermoproteota* and harboured *amoA* genes. These have also been identified in high cell densities in other corals species [14,104], suggesting that archaea participate in nitrogen cycling in a range of corals.

Nitrogen can also be assimilated by microbes in the coral holobiont possessing nitrate reductases. We identified complete nitrogen assimilation and dissimilation modules in MAGs from different microbial lineages in both coral species (Figure 3a and b). As the coral skeleton turns anoxic rapidly in darkness [95], denitrification and dissimilatory nitrate reduction (DNRA) activity have been hypothesised to be upregulated [14,105]. With conditions, including, near anoxia and limited nitrate availability in darkness, tailored for DNRA to outcompete denitrification, it was no surprise that only 3 MAGs recovered from *P. lutea* and *I palifera* harboured complete denitrification pathway. DNRA presents a significant N retention mechanism under dark conditions and can function as the principal pathway contributing to ammonia availability for assimilation in the coral [106].

Conclusion

By applying genome-resolved metagenomics to the coral skeleton, we provide a comprehensive genomic view of the diversity and functional potential of the prokaryotic component of the skeletal microbiome. This study expands and enriches our understanding of the coral skeletal microbiome's role in holobiont functioning. Also, by undertaking a genome-centric study, we identified how the skeletal microbiome members harbour an arsenal of stress mediators, including DMSP synthesis and metabolism genes. These prokaryotic microbes have a diverse array of ELPs to establish symbiosis with the coral host and/or other eukaryotes in the coral holobiont. Importantly, we show that skeletal microbiomes from *P. lutea* and *I. palifera* have the potential to contribute to the nitrogen and sulphur cycling budget of the host. We provide a framework for future studies focused on identifying the key members of the skeletal holobiont and ascertaining their role in coral

health, and how the skeletal microbiome functionally responds when the corals are under stress.

Acknowledgements

This work was funded through the Australian Research Council grant DP200101613 (to HV, LLB, MM and MK), the Faculty of Science (University of Melbourne, to HV), and the Holsworth Wildlife endowment (to FR). MK acknowledges support from the Gordon and Betty Moore Foundation through grant no. GBMF9206 (https://doi.org/10.37807/GBMF9206). MM acknowledges support from NOAA CRCP NA19NOS4820132.

Author contributions

K.T and H.V contributed to the conceptual development of the work and manuscript. F.R and J.C conducted the experiments. K.T conducted the data analysis and wrote the first draft. All authors contributed to the final edited version of the manuscript.

Conflict of Interest

On behalf of all authors, the corresponding author states that there is no conflict of interest.

References

1. Bourne DG, Webster NS. Coral Reef Bacterial Communities. In: Rosenberg E, DeLong EF, Lory S, Stackebrandt E, Thompson F, editors. The Prokaryotes: Prokaryotic Communities and Ecophysiology. Berlin, Heidelberg: Springer Berlin Heidelberg; 2013. p. 163–87.

2. Blackall LL, Wilson B, van Oppen MJH. Coral-the world's most diverse symbiotic ecosystem. Mol Ecol. 2015;24:5330–47.

3. van Oppen MJH, Blackall LL. Coral microbiome dynamics, functions and design in a changing world. Nat Rev Microbiol. 2019;17:557–67.

4. Reshef L, Koren O, Loya Y, Zilber-Rosenberg I, Rosenberg E. The coral probiotic hypothesis. Environ Microbiol. Wiley; 2006;8:2068–73.

5. Peixoto RS, Sweet M, Villela HDM, Cardoso P, Thomas T, Voolstra CR, et al. Coral Probiotics: Premise, Promise, Prospects. Annu Rev Anim Biosci. 2021;9:265–88.

6. Rosado PM, Leite DCA, Duarte GAS, Chaloub RM, Jospin G, da Rocha UN, et al. Marine probiotics: increasing coral resistance to bleaching through microbiome manipulation. ISME J. Nature Publishing Group; 2018;13:921–36.

7. Krediet CJ, Ritchie KB, Paul VJ, Teplitski M. Coral-associated micro-organisms and their roles in promoting coral health and thwarting diseases. Proc Biol Sci. 2013;280:20122328.

8. Peixoto RS, Rosado PM, Leite DC de A, Rosado AS, Bourne DG. Beneficial Microorganisms for Corals (BMC): Proposed Mechanisms for Coral Health and Resilience. Front Microbiol. 2017;8:341.

9. Tandon K, Lu C-Y, Chiang P-W, Wada N, Yang S-H, Chan Y-F, et al. Comparative genomics: Dominant coral-bacterium Endozoicomonas acroporae metabolizes dimethylsulfoniopropionate (DMSP). ISME J. 2020;14:1290–303.

10. Bourne D, Iida Y, Uthicke S, Smith-Keune C. Changes in coral-associated microbial communities during a bleaching event. ISME J. 2008;2:350–63.

11. Kimes NE, Van Nostrand JD, Weil E, Zhou J, Morris PJ. Microbial functional structure of Montastraea faveolata, an important Caribbean reef-building coral, differs between healthy and yellow-band diseased colonies. Environ Microbiol. 2010;12:541–56.

12. O’Brien PA, Smith HA, Fallon S, Fabricius K, Willis BL, Morrow KM, et al. Elevated CO2 Has Little Influence on the Bacterial Communities Associated With the pH-Tolerant Coral, Massive Porites spp. Front Microbiol. 2018;9:2621.

13. Ricci F, Rossetto Marcelino V, Blackall LL, Kühl M, Medina M, Verbruggen H. Beneath the surface: community assembly and functions of the coral skeleton microbiome. Microbiome. 2019;7:159.

14. Siboni N, Ben-Dov E, Sivan A, Kushmaro A. Global distribution and diversity of coral-associated Archaea and their possible role in the coral holobiont nitrogen cycle. Environ Microbiol. Wiley; 2008;10:2979–90.

15. Kellogg CA. Tropical Archaea: diversity associated with the surface microlayer of corals. Mar Ecol Prog Ser. Inter-Research Science Center; 2004;273:81–8.

16. Marcelino VR, van Oppen MJ, Verbruggen H. Highly structured prokaryote communities exist within the skeleton of coral colonies. ISME J. 2018;12:300–3.

17. Marcelino VR, Verbruggen H. Multi-marker metabarcoding of coral skeletons reveals a rich microbiome and diverse evolutionary origins of endolithic algae. Sci Rep. 2016;6:31508.

18. Yang S-H, Lee STM, Huang C-R, Tseng C-H, Chiang P-W, Chen C-P, et al. Prevalence of potential nitrogen-fixing, green sulfur bacteria in the skeleton of reef-building coral *Isopora*

palifera [Internet]. Limnology and Oceanography. 2016. p. 1078–86. Available from: http://dx.doi.org/10.1002/lno.10277

19. Yang S-H, Tandon K, Lu C-Y, Wada N, Shih C-J, Hsiao SS-Y, et al. Metagenomic, phylogenetic, and functional characterization of predominant endolithic green sulfur bacteria in the coral Isopora palifera. Microbiome. 2019;7:3.

20. Ricci F, Fordyce A, Leggat W, Blackall LL, Ainsworth T, Verbruggen H. Multiple techniques point to oxygenic phototrophs dominating the Isopora palifera skeletal microbiome. Coral Reefs. 2021;40:275–82.

21. Ricci F, Tandon K, Black JR, Lê Cao K-A, Blackall LL, Verbruggen H. Host Traits and Phylogeny Contribute to Shaping Coral-Bacterial Symbioses. mSystems. 2022;7:e0004422.

22. Kendrick B, Risk MJ, Michaelides J, Bergman K. Amphibious Microborers: Bioeroding Fungi Isolated from Live Corals. Bull Mar Sci. 1982;32:862–7.

23. Bentis CJ, Kaufman L, Golubic S. Endolithic fungi in reef-building corals (Order : Scleractinia) are common, cosmopolitan, and potentially pathogenic. Biol Bull. 2000;198:254–60.

24. Golubic S, Radtke G, Le Campion-Alsumard T. Endolithic fungi in marine ecosystems. Trends Microbiol. 2005;13:229–35.

25. Sweet MJ, Croquer A, Bythell JC. Bacterial assemblages differ between compartments within the coral holobiont. Coral Reefs. 2011;30:39–52.

26. Galindo-Martínez CT, Weber M, Avila-Magaña V, Enríquez S, Kitano H, Medina M, et al. The role of the endolithic alga Ostreobium spp. during coral bleaching recovery. Sci Rep. 2022;12:2977.

27. Sangsawang L, Casareto BE, Ohba H, Vu HM, Meekaew A, Suzuki T, et al. 13C and 15N assimilation and organic matter translocation by the endolithic community in the massive coral Porites lutea. R Soc Open Sci. 2017;4:171201.

28. Tandon K, Pasella MM, Iha C, Ricci F, Hu J, O'Kelly CJ, et al. Every refuge has its price: Ostreobium as a model for understanding how algae can live in rock and stay in business. Semin Cell Dev Biol [Internet]. 2022; Available from: http://dx.doi.org/10.1016/j.semcdb.2022.03.010

29. Pernice M, Raina J-B, Rädecker N, Cárdenas A, Pogoreutz C, Voolstra CR. Down to the bone: the role of overlooked endolithic microbiomes in reef coral health. ISME J. Nature Publishing Group; 2019;14:325–34.

30. Olson ND, Ainsworth TD, Gates RD, Takabayashi M. Diazotrophic bacteria associated with Hawaiian Montipora corals: Diversity and abundance in correlation with symbiotic dinoflagellates. J Exp Mar Bio Ecol. 2009;371:140–6.

31. Lema KA, Bourne DG, Willis BL. Onset and establishment of diazotrophs and other bacterial associates in the early life history stages of the coral Acropora millepora. Mol Ecol.

2014;23:4682–95.

32. Lema KA, Willis BL, Bourne DG. Amplicon pyrosequencing reveals spatial and temporal consistency in diazotroph assemblages of the Acropora millepora microbiome. Environ Microbiol. 2014;16:3345–59.

33. Crossland CJ, Barnes DJ. Acetylene reduction by coral skeletons. Limnol Oceanogr. Wiley; 1976;21:153–6.

34. Williams WM, Viner AB, Broughton WJ. Nitrogen fixation (acetylene reduction) associated with the living coral Acropora variabilis. Mar Biol. Springer Nature; 1987;94:531–5.

35. Cai L, Zhou G, Tian R-M, Tong H, Zhang W, Sun J, et al. Metagenomic analysis reveals a green sulfur bacterium as a potential coral symbiont. Sci Rep. 2017;7:9320.

36. Cárdenas A, Raina J-B, Pogoreutz C, Rädecker N, Bougoure J, Guagliardo P, et al. Greater functional diversity and redundancy of coral endolithic microbiomes align with lower coral bleaching susceptibility. ISME J [Internet]. 2022; Available from: http://dx.doi.org/10.1038/s41396-022-01283-y

37. Ewels P, Magnusson M, Lundin S, Käller M. MultiQC: summarize analysis results for multiple tools and samples in a single report. Bioinformatics. 2016;32:3047–8.

38. Bolger AM, Lohse M, Usadel B. Trimmomatic: a flexible trimmer for Illumina sequence data. Bioinformatics. 2014;30:2114–20.

39. Langmead B, Salzberg SL. Fast gapped-read alignment with Bowtie 2. Nat Methods. 2012;9:357–9.

40. Li H, Handsaker B, Wysoker A, Fennell T, Ruan J, Homer N, et al. The Sequence Alignment/Map format and SAMtools. Bioinformatics. 2009;25:2078–9.

41. Li D, Luo R, Liu C-M, Leung C-M, Ting H-F, Sadakane K, et al. MEGAHIT v1.0: A fast and scalable metagenome assembler driven by advanced methodologies and community practices. Methods. 2016;102:3–11.

42. Alneberg J, Bjarnason BS, de Bruijn I, Schirmer M, Quick J, Ijaz UZ, et al. CONCOCT: Clustering cONtigs on COverage and ComposiTion [Internet]. arXiv [q-bio.GN]. 2013. Available from: http://arxiv.org/abs/1312.4038

43. Wu Y-W, Simmons BA, Singer SW. MaxBin 2.0: an automated binning algorithm to recover genomes from multiple metagenomic datasets. Bioinformatics. 2016;32:605–7.

44. Kang DD, Li F, Kirton E, Thomas A, Egan R, An H, et al. MetaBAT 2: an adaptive binning algorithm for robust and efficient genome reconstruction from metagenome assemblies. PeerJ. 2019;7:e7359.

45. Uritskiy GV, DiRuggiero J, Taylor J. MetaWRAP—a flexible pipeline for genome-resolved metagenomic data analysis. Microbiome. BioMed Central; 2018;6:1–13.

46. Kang DD, Froula J, Egan R, Wang Z. MetaBAT, an efficient tool for accurately reconstructing single genomes from complex microbial communities. PeerJ. 2015;3:e1165.

47. Olm MR, Brown CT, Brooks B, Banfield JF. dRep: a tool for fast and accurate genomic comparisons that enables improved genome recovery from metagenomes through de-replication. ISME J. 2017;11:2864–8.

48. Parks DH, Imelfort M, Skennerton CT, Hugenholtz P, Tyson GW. CheckM: assessing the quality of microbial genomes recovered from isolates, single cells, and metagenomes [Internet]. PeerJ. 2015. Available from: http://dx.doi.org/10.7287/peerj.preprints.554

49. von Meijenfeldt FAB, Arkhipova K, Cambuy DD, Coutinho FH, Dutilh BE. Robust taxonomic classification of uncharted microbial sequences and bins with CAT and BAT. Genome Biol. 2019;20:217.

50. Chaumeil P-A, Mussig AJ, Hugenholtz P, Parks DH. GTDB-Tk: a toolkit to classify genomes with the Genome Taxonomy Database. Bioinformatics [Internet]. 2019; Available from: http://dx.doi.org/10.1093/bioinformatics/btz848

51. Jain C, Rodriguez-R LM, Phillippy AM, Konstantinidis KT, Aluru S. High throughput ANI analysis of 90K prokaryotic genomes reveals clear species boundaries. Nat Commun. 2018;9:5114.

52. Matsen FA, Kodner RB, Armbrust EV. pplacer: linear time maximum-likelihood and Bayesian phylogenetic placement of sequences onto a fixed reference tree. BMC Bioinformatics. 2010;11:538.

53. Bushnell B. BBMap: a fast, accurate, splice-aware aligner [Internet]. Lawrence Berkeley National Lab.(LBNL), Berkeley, CA (United States); 2014. Available from: https://www.osti.gov/biblio/1241166

54. Ihaka R, Gentleman R. R: A Language for Data Analysis and Graphics. J Comput Graph Stat. Taylor & Francis; 1996;5:299–314.

55. Wickham H. ggplot2. Wiley Interdiscip Rev Comput Stat. Wiley; 2011;3:180–5.

56. Minh BQ, Schmidt HA, Chernomor O, Schrempf D, Woodhams MD, von Haeseler A, et al. IQ-TREE 2: New Models and Efficient Methods for Phylogenetic Inference in the Genomic Era. Mol Biol Evol. 2020;37:1530–4.

57. Letunic I, Bork P. Interactive Tree Of Life (iTOL) v4: recent updates and new developments. Nucleic Acids Res. 2019;47:W256–9.

58. Hyatt D, Chen G-L, Locascio PF, Land ML, Larimer FW, Hauser LJ. Prodigal: prokaryotic gene recognition and translation initiation site identification. BMC Bioinformatics. 2010;11:119.

59. Seemann T. Prokka: rapid prokaryotic genome annotation. Bioinformatics. Oxford University Press (OUP); 2014;30:2068–9.

60. Jones P, Binns D, Chang H-Y, Fraser M, Li W, McAnulla C, et al. InterProScan 5: genome-

scale protein function classification. Bioinformatics. 2014;30:1236–40.

61. Zhou Z, Tran PQ, Breister AM, Liu Y, Kieft K, Cowley ES, et al. METABOLIC: high-throughput profiling of microbial genomes for functional traits, metabolism, biogeochemistry, and community-scale functional networks. Microbiome. 2022;10:33.

62. Kolde R. pheatmap: Pretty Heatmaps. R package version 1.0. 12. R Packag version 1 0. 2019;8.

63. Jernigan KK, Bordenstein SR. Tandem-repeat protein domains across the tree of life. PeerJ. 2015;3:e732.

64. van Oppen MJH, Oliver JK, Putnam HM, Gates RD. Building coral reef resilience through assisted evolution. Proc Natl Acad Sci U S A. 2015;112:2307–13.

65. Damjanovic K, van Oppen MJH, Menéndez P, Blackall LL. Experimental Inoculation of Coral Recruits With Marine Bacteria Indicates Scope for Microbiome Manipulation in Acropora tenuis and Platygyra daedalea. Front Microbiol. 2019;10:1702.

66. Doering T, Wall M, Putchim L, Rattanawongwan T, Schroeder R, Hentschel U, et al. Towards enhancing coral heat tolerance: a “microbiome transplantation” treatment using inoculations of homogenized coral tissues. Microbiome. 2021;9:102.

67. Santoro EP, Borges RM, Espinoza JL, Freire M, Messias CSMA, Villela HDM, et al. Coral microbiome manipulation elicits metabolic and genetic restructuring to mitigate heat stress and evade mortality. Sci Adv [Internet]. 2021;7. Available from: http://dx.doi.org/10.1126/sciadv.abg3088

68. Ricci F, Tandon K, Moßhammer M, Cho EH-J, Blackall LL, Kühl M, et al. Fine-scale mapping of physicochemical and microbial landscapes clarifies the spatial structure of the coral skeleton microbiome. 2022 [cited 2022 Jun 12]; Available from: https://www.researchsquare.com/article/rs-1735748/v1

69. Robbins SJ, Singleton CM, Chan CX, Messer LF, Geers AU, Ying H, et al. A genomic view of the reef-building coral Porites lutea and its microbial symbionts. Nat Microbiol. 2019;4:2090–100.

70. Góes-Neto A, Marcelino VR, Verbruggen H, da Silva FF. Biodiversity of endolithic fungi in coral skeletons and other reef substrates revealed with 18S rDNA metabarcoding. Coral Reefs [Internet]. Springer; 2020; Available from: https://link.springer.com/article/10.1007/s00338-019-01880-y

71. Martyn JE, Gomez-Valero L, Buchrieser C. The evolution and role of eukaryotic-like domains in environmental intracellular bacteria: the battle with a eukaryotic cell. FEMS Microbiol Rev [Internet]. 2022; Available from: http://dx.doi.org/10.1093/femsre/fuac012

72. Jernigan KK, Bordenstein SR. Ankyrin domains across the Tree of Life. PeerJ 2: e264. 2014.

73. Li J, Mahajan A, Tsai M-D. Ankyrin repeat: a unique motif mediating protein-protein

interactions. Biochemistry. 2006;45:15168–78.

74. Nguyen MTHD, Liu M, Thomas T. Ankyrin-repeat proteins from sponge symbionts modulate amoebal phagocytosis. Mol Ecol. 2014;23:1635–45.

75. Sweet M, Villela H, Keller-Costa T, Costa R, Romano S, Bourne DG, et al. Insights into the Cultured Bacterial Fraction of Corals. mSystems. 2021;6:e0124920.

76. Horn M. Chlamydiae as symbionts in eukaryotes. Annu Rev Microbiol. 2008;62:113–31.

77. Neer EJ, Schmidt CJ, Nambudripad R, Smith TF. The ancient regulatory-protein family of WD-repeat proteins. Nature. 1994;371:297–300.

78. Hu X-J, Li T, Wang Y, Xiong Y, Wu X-H, Zhang D-L, et al. Prokaryotic and Highly-Repetitive WD40 Proteins: A Systematic Study. Sci Rep. 2017;7:10585.

79. Tandon K, Chiou Y-J, Yu S-P, Hsieh HJ, Lu C-Y, Hsu M-T, et al. Microbiome Restructuring: Dominant Coral Bacterium Endozoicomonas Species Respond Differentially to Environmental Changes. mSystems. 2022;e0035922.

80. Thomas T, Rusch D, DeMaere MZ, Yung PY, Lewis M, Halpern A, et al. Functional genomic signatures of sponge bacteria reveal unique and shared features of symbiosis. ISME J. 2010;4:1557–67.

81. Reynolds D, Thomas T. Evolution and function of eukaryotic-like proteins from sponge symbionts. Mol Ecol. 2016;25:5242–53.

82. Robbins SJ, Song W, Engelberts JP, Glasl B, Slaby BM, Boyd J, et al. A genomic view of the microbiome of coral reef demosponges. ISME J. 2021;15:1641–54.

83. Li Jie, Zou Yiyang, Yang Jian, Li Qiqi, Bourne David G., Sweet Michael, et al. Cultured Bacteria Provide Insight into the Functional Potential of the Coral-Associated Microbiome. mSystems. American Society for Microbiology; 0:e00327-22.

84. Pogoreutz C, Oakley CA, Rädecker N, Cárdenas A, Perna G, Xiang N, et al. Coral holobiont cues prime Endozoicomonas for a symbiotic lifestyle. ISME J [Internet]. 2022; Available from: http://dx.doi.org/10.1038/s41396-022-01226-7

85. Edqvist PJ, Bröms JE, Betts HJ, Forsberg A, Pallen MJ, Francis MS. Tetratricopeptide repeats in the type III secretion chaperone, LcrH: their role in substrate binding and secretion. Mol Microbiol. 2006;59:31–44.

86. Cerveny L, Straskova A, Dankova V, Hartlova A, Ceckova M, Staud F, et al. Tetratricopeptide repeat motifs in the world of bacterial pathogens: role in virulence mechanisms. Infect Immun. 2013;81:629–35.

87. Vanwonterghem I, Webster NS. Coral Reef Microorganisms in a Changing Climate. iScience. 2020;23:100972.

88. D Ainsworth T, Krause L, Bridge T, Torda G, Raina J-B, Zakrzewski M, et al. The coral core microbiome identifies rare bacterial taxa as ubiquitous endosymbionts. ISME J.

2015;9:2261–74.

89. Raina J-B, Tapiolas D, Willis BL, Bourne DG. Coral-associated bacteria and their role in the biogeochemical cycling of sulfur. Appl Environ Microbiol. 2009;75:3492–501.

90. Raina J-B, Dinsdale EA, Willis BL, Bourne DG. Do the organic sulfur compounds DMSP and DMS drive coral microbial associations? Trends Microbiol. 2010;18:101–8.

91. Frade PR, Schwaninger V, Glasl B, Sintes E, Hill RW, Simó R, et al. Dimethylsulfoniopropionate in corals and its interrelations with bacterial assemblages in coral surface mucus. Environ Chem. CSIRO PUBLISHING; 2015;13:252–65.

92. Hernandez-Agreda A, Gates RD, Ainsworth TD. Defining the Core Microbiome in Corals' Microbial Soup. Trends Microbiol. 2017;25:125–40.

93. Kuek FWI, Motti CA, Zhang J, Cooke IR, Todd JD, Miller DJ, et al. DMSP production by coral-associated bacteria. Front Mar Sci [Internet]. Frontiers Media SA; 2022;9. Available from: https://www.frontiersin.org/articles/10.3389/fmars.2022.869574/full

94. Williams BT, Cowles K, Bermejo Martínez A, Curson ARJ, Zheng Y, Liu J, et al. Bacteria are important dimethylsulfoniopropionate producers in coastal sediments. Nat Microbiol. 2019;4:1815–25.

95. Kühl M, Holst G, Larkum AWD, Ralph PJ. Imaging of oxygen dynamics within the endolithic algal community of the massive coral Porites Lobata(1). J Phycol. Wiley; 2008;44:541–50.

96. Yuen YS, Yamazaki SS, Baird AH, Nakamura T, Yamasaki H. Sulfate-reducing bacteria in the skeleton of the massive coral *Goniastrea aspera* from the great barrier reef. Galaxea, Journal of Coral Reef Studies. 2013;15:154–9.

97. Sánchez-Quinto A, Falcón LI. Metagenome of Acropora palmata coral rubble: Potential metabolic pathways and diversity in the reef ecosystem. PLoS One. 2019;14:e0220117.

98. Chen Y-H, Yang S-H, Tandon K, Lu C-Y, Chen H-J, Shih C-J, et al. Potential syntrophic relationship between coral-associated Prosthecochloris and its companion sulfate-reducing bacterium unveiled by genomic analysis. Microb Genom [Internet]. 2021;7. Available from: http://dx.doi.org/10.1099/mgen.0.000574

99. Rädecker N, Pogoreutz C, Voolstra CR, Wiedenmann J, Wild C. Nitrogen cycling in corals: the key to understanding holobiont functioning? Trends Microbiol. 2015;23:490–7.

100. Webb KL, DuPaul WD, Wiebe W, Sottile W, Johannes RE. Enewetak (Eniwetok) Atoll: Aspects of the nitrogen cycle on a coral reef1. Limnol Oceanogr. Wiley; 1975;20:198–210.

101. Lesser MP, Mazel CH, Gorbunov MY, Falkowski PG. Discovery of symbiotic nitrogen-fixing cyanobacteria in corals. Science. 2004;305:997–1000.

102. Shashar N, Cohen Y, Loya Y, Sar N. Nitrogen fixation (acetylene reduction) in stony corals: evidence for coral-bacteria interactions. Mar Ecol Prog Ser. Inter-Research Science Center; 1994;111:259–64.

103. Beman JM, Roberts KJ, Wegley L, Rohwer F, Francis CA. Distribution and diversity of archaeal ammonia monooxygenase genes associated with corals. Appl Environ Microbiol. 2007;73:5642–7.

104. Siboni N, Ben-Dov E, Sivan A, Kushmaro A. Geographic specific coral-associated ammonia-oxidizing archaea in the northern Gulf of Eilat (Red Sea). Microb Ecol. 2012;64:18–24.

105. Shashar N, Cohen Y, Loya Y. Extreme Diel Fluctuations of Oxygen in Diffusive Boundary Layers Surrounding Stony Corals. Biol Bull. 1993;185:455–61.

106. Glaze TD, Erler DV, Siljanen HMP. Microbially facilitated nitrogen cycling in tropical corals. ISME J. 2022;16:68–77.

Figure Legends

Figure 1. Phylogenetic trees of Metagenome-assembled genomes (MAGs) recovered from *P. lutea* and *I. palifera* skeleton**.** a) 376 bacterial MAGs, with genome completeness, GC content, and genes-of-interest and b) 17 archaeal MAGs with the presence of ammonia oxidising gene *AmoA.* The phylogenetic tree was constructed using a concatenated alignment of 120 bacterial and 122 archaeal marker genes, respectively. Taxonomic annotation of bacterial MAGs (counterclockwise): *Proteobacteria, Desulfobacterota,* DSWW01, *Desulfobacteriota*_F, *Nitrospinota*, *Bdellovibrionota*, *Desulfobacterota*_B, *Myxococcota*, SAR324. *Bdellovibrionota*, *Acidibacteriota*, *Bacteroidota*, *Calditrichota*, SM23-31, AABM5-125-54, *Marinosomatota*, Zixibacteria, Gemmatimondota, *Fibriobacterota*, *Elusimicrobiota*, *Omnitrophota, Sumerlaeota, Planctomycetota, Verrucomicrobiota, Chlamydiota, Spirochaetota, Firmicutes*_A*, Firmicutes, Fimicutes*_H*, Firmicutes*_G*, Cyanobacteria, Chloroflexota, Patescibacteria, Actinobacteriota, Bipolaricaulota.*

Figure 2. Heatmap representing different categories of Eukaryotic-like proteins (ELPs) from **a)** *P. lutea*and **b)** *I. palifera* skeletal MAGs. Bar plots represent the total count of ELPs in a MAG. Numbers in the ARP column denote the count of Ankyrin repeat proteins in MAGs which devote >0.2% of total genes to ARPs, suggesting a potentially host-associated lifestyle. Detailed information about these MAGs is available in the Supplementary data file.

Figure 3 Heatmap of KEGG-based nitrogen, sulphur and anoxygenic photosynthesis modules in a) *P. lutea* and b) *I. palifera* MAGs, with the representation of their taxonomic classes. MAGs with at least one module >75% complete are shown here.

Figure 1

Click here to access/download;Figure;Figure 1.pdf

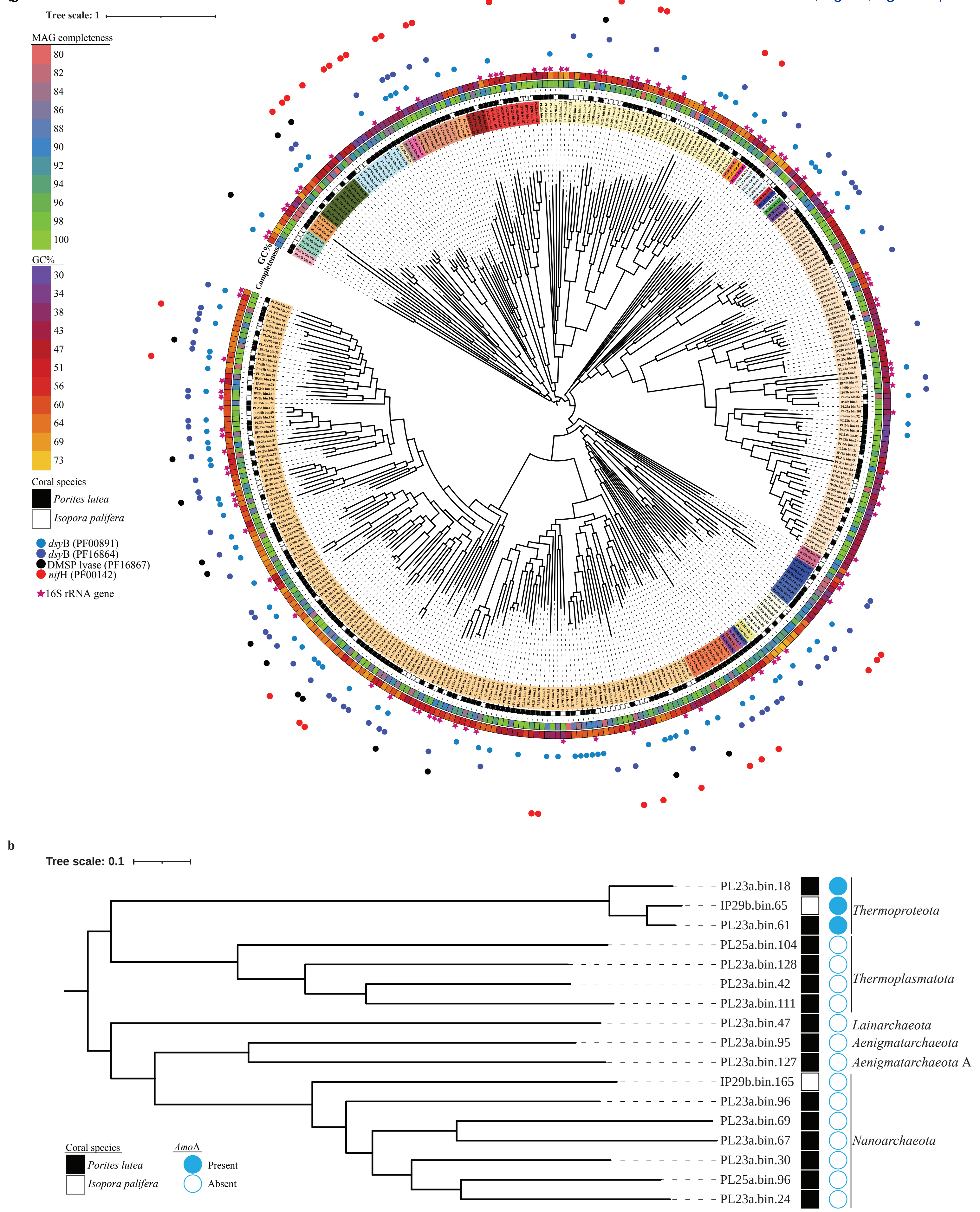

Figure 2

Click here to access/download;Figure;Figure 2.pdf

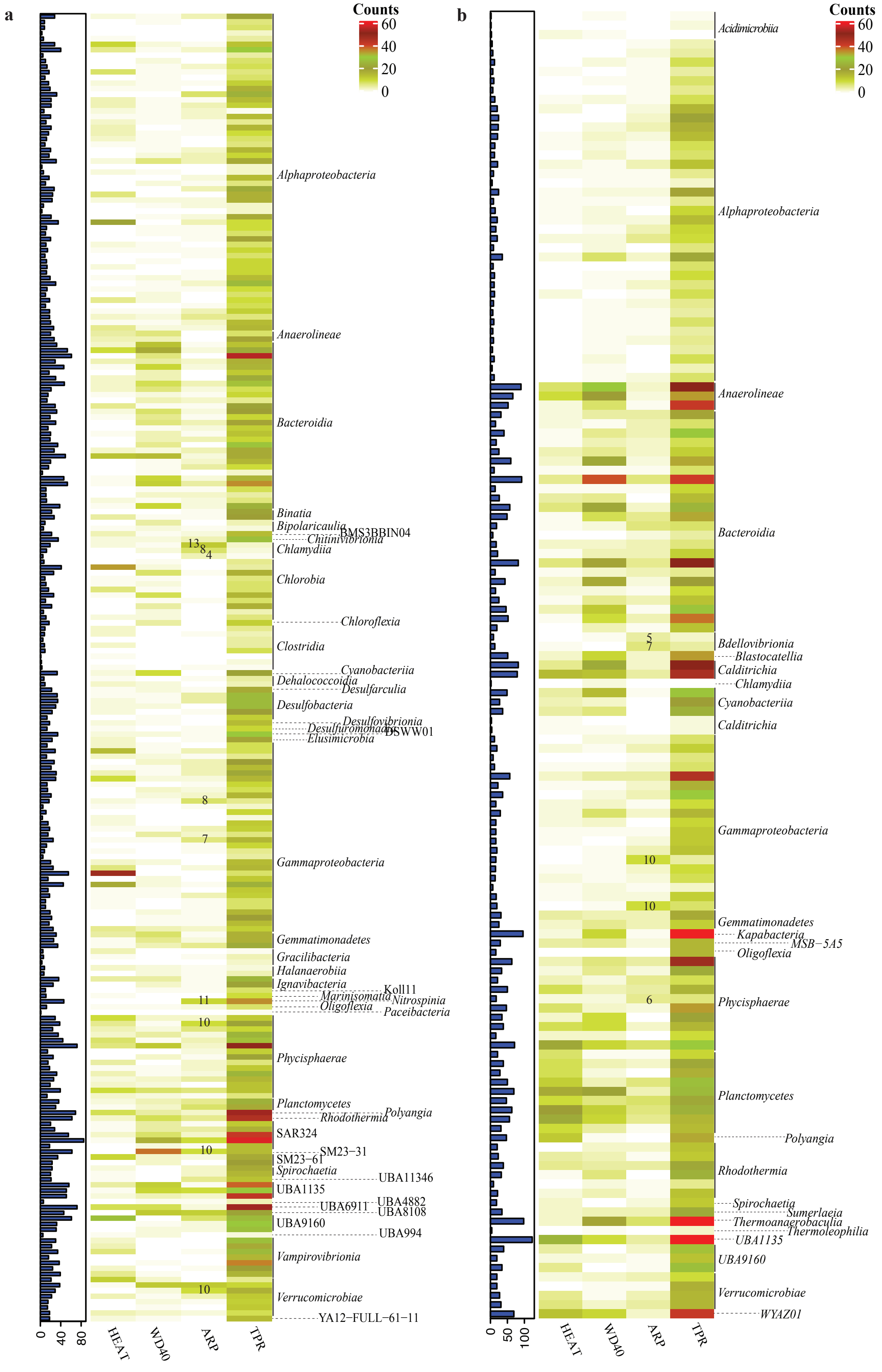

Figure 3

Click here to access/download;Figure;Figure 3.pdf

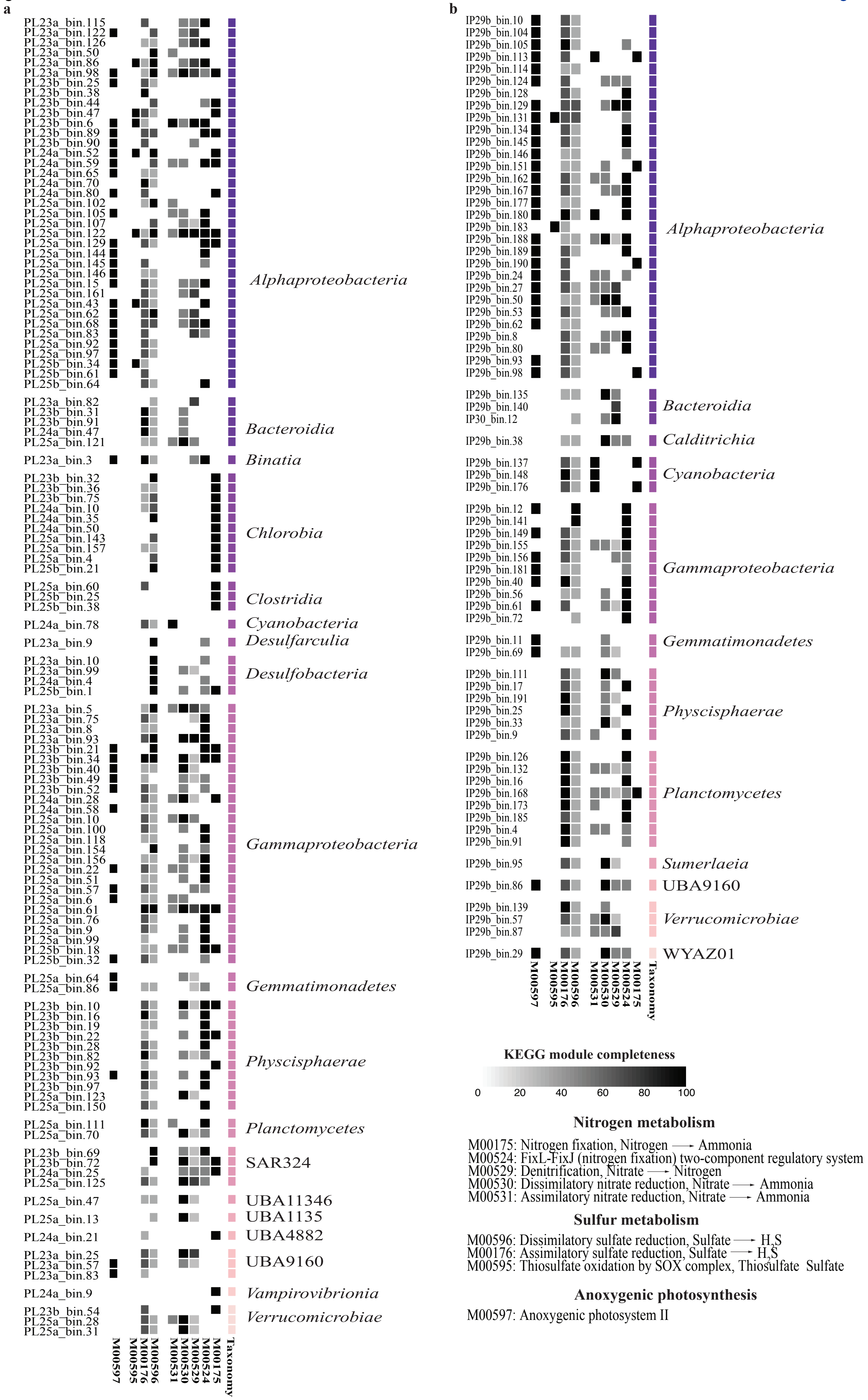

Click here to access/download
Supplementary Material
Tandon K et al Supplementary Material.docx

Click here to access/download
Supplementary Material
Figure S1_Supplementary Material.pdf

Click here to access/download
Supplementary Material
Figure S2_Supplementary Material.pdf

Click here to access/download
Supplementary Material
Figure S3_Supplementary Material.pdf

Cover letter Click here to access/download;Personal Cover;Cover Letter.docx

Dear *GigaScience* editorial board,

On behalf of me and my co-authors, please find enclosed our manuscript entitled: "Genomic view of the diversity and functional role of archaea and bacteria in the skeleton of reef-building corals *Porites lutea* and *Isopora palifera*" for consideration for publication in *GigaScience* as a full-length research article.

Corals harbour highly distinct and diverse microbiomes in their different compartments and it is important to study them in greater detail to understand the intricate relation of coral holobiont members. In recent years, we have gained comprehensive knowledge on the coral-tissue microbiome and its role in coral health, but the microbiome in coral skeletons has been less characterized and has remained enigmatic. Currently, there are only a handful of studies relying primarily on metabarcoding approaches to characterize the coral-skeletal microbiome. This approach makes it impossible to gain knowledgeable insights into the functional potential of the coral skeletal microbiome, rendering its role in coral health elusive. A rapid decline in global coral reefs has spurred extensive research on mitigation strategies like the development of coral probiotics. However, until we gain detailed insights into the less-studied coral skeletal microbiome, these endeavours will remain difficult to achieve.

In this study, we applied genome-resolved metagenomics to provide a comprehensive genome-scale view of the coral skeletal microbiome. This study provides an unprecedented resource of ~400 high-quality archaeal and bacterial metagenome-assembled genomes (MAGs) spanning 37 phyla and 57 microbial classes from the skeleton of two reef-building corals *Porites lutea* and *Isopora palifera*. Many skeletal microbiome members were identified to encode oxidative stress-response genes e.g dimethylsulfoniopropionate synthase and lyases. In addition, we also identified that skeletal microbiome MAGs encoded more than 20 genes of eukaryotic origin, suggesting diverse mechanisms these microbes can use to establish symbiosis with coral hosts and other microeukaryotes living in the skeleton. Furthermore, nitrogen metabolism, particularly nitrogen fixation, was widespread along with the ability to cycle sulphur compounds, depicting the potential of the skeletal microbiome to contribute to the nitrogen and sulphur cycling budget of the host.

This manuscript is our original research; a preprint version of this manuscript was published in ResearchSquare with the doi: 10.21203/rs.3.rs-1890655/v1. The manuscript has not been submitted for publication elsewhere while under consideration in *GigaScience* All authors declare that they have no conflict of interest.

We believe this manuscript contributes important knowledge to the field of coral biology and brings focus to an important but less-studied coral skeletal microbiome and its functional role. Therefore, this manuscript is suitable for the scope of *GigaScience* and its readers.

To this end, we propose the following reviewers for this work based on their research focus and experience

1. Prof. Rebecca Vega Thurber (Oregon State University, USA), Rebecca.Vega-Thurber@oregonstate.edu

2. Dr Jean Baptiste Raina (University of Technology, Sydney, Australia) jean-baptiste.raina@uts.edu.au
3. Dr. Claudia Pogoreutz (École Polytechnique Fédérale de Lausanne) claudia.pogoreutz@epfl.ch
4. Dr Raquel Peixoto (King Abdullah University, Saudi Arabia) raquel.peixoto@kaust.edu.sa
5. Dr Anna Roik (Helmholtz Institute of Functional Marine Biodiveristy) anna.roik@hfimb.de

We look forward to hearing from you.

Sincerely,

Dr Kshitij Tandon

Research Fellow, School of BioSciences,

University of Melbourne, Melbourne, VIC, Australia

Email: k.tandon@unimelb.edu.au
