# Supplementary material for: Genomic view of the diversity and functional role of archaea and bacteria in the skeleton of the reef-building corals Porites lutea and Isopora palifera
Source: Gigascience. 2023 Jan 23;12:giac127. doi: 10.1093/gigascience/giac127 (PMC9868349; doi:10.1093/gigascience/giac127)
Supplement: giac127_Supplemental_Files [file giac127_supplemental_files.zip › Tandon K et al Supplementary Material.docx]

**Genomic view of the diversity and functional role of archaea and bacteria in the skeleton of the reef-building corals *Porites lutea* and *Isopora palifera***

Kshitij Tandon^1#^, Francesco Ricci^1,2^, Joana Costa^1^, Mónica Medina^3^, Michael Kühl^4^, Linda L. Blackall^1^, Heroen Verbruggen^1^

^1^ School of BioSciences, University of Melbourne, Parkville 3010, Australia

^2^Biological, Earth and Environmental Sciences, The University of New South Wales, Kensington, NSW 2052, Australia

^3^ Department of Biology, Pennsylvania State University, University Park, PA 16802, USA

^4^ Marine Biological Section, Department of Biology, University of Copenhagen, Strandpromenaden 5, DK-3000 Helsingør, Denmark

^#^Corresponding author: Kshitij Tandon

Corresponding author email: k.tandon@unimelb.edu.au

**Supplementary Material**

**Supplementary data File**: Dataset with details of all the MAGs recovered in this work, including genome statistics, genes of interest and accession numbers.

**Supplementary Figure S1.** Barplots depicting the metagenome bins recovered after each step. Only High-quality bins were used for performing all the analysis.

**Supplementary Figure S2.** Boxplot for P. lutea and I. palifera MAGs completeness and contamination stats.

**Supplementary Figure S3.** Stacked barplots for Relative abundance (>1%) of MAGs taxonomic classes for *P. lutea* and *I. palifera*.  p_***Aenigmatarchaeota***, p_***Nanoarchaeota***, p_***Thermoproteota*** represents the Archaea domain.

**Supplementary Figure S4.** Heatmaps representing bacterial MAGs from a) *P. lutea* and b) I. *palifera* with at least one KEGG module >75% complete. MAGs are annotated at Class level.
