# Supplementary figures and images for: Genomic view of the diversity and functional role of archaea and bacteria in the skeleton of the reef-building corals Porites lutea and Isopora palifera
Source: Gigascience. 2023 Jan 23;12:giac127. doi: 10.1093/gigascience/giac127 (PMC9868349; doi:10.1093/gigascience/giac127)

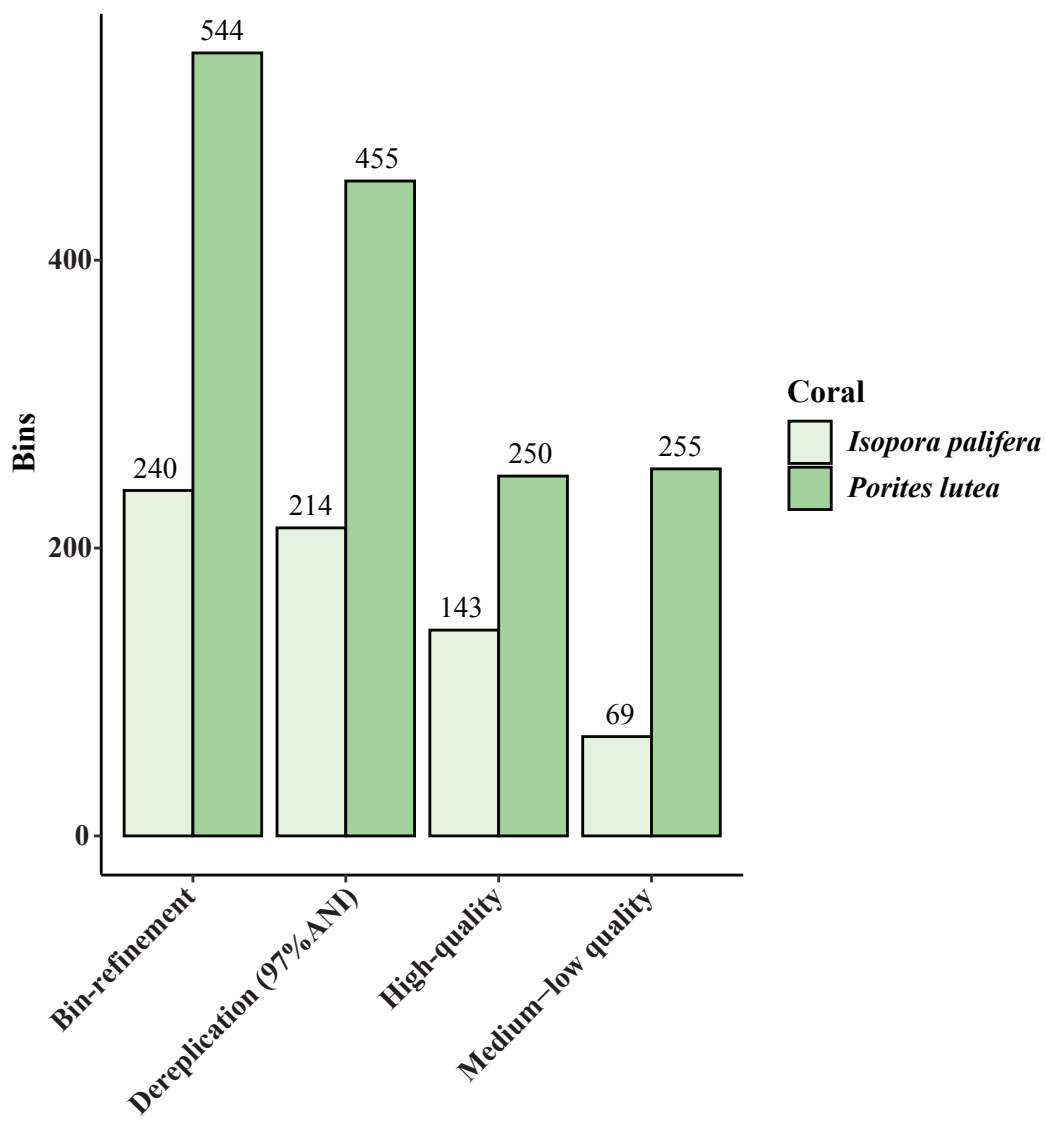

Supplement: giac127_Supplemental_Files [file giac127_supplemental_files.zip › Figure S1_Supplementary Material.pdf]

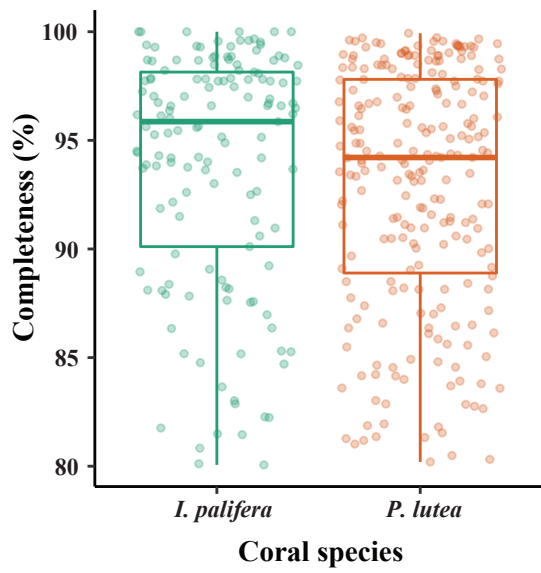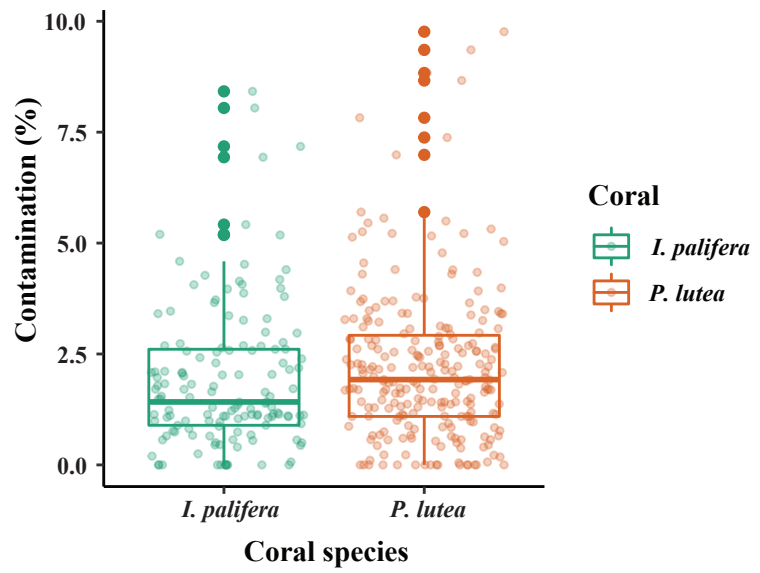

Supplement: giac127_Supplemental_Files [file giac127_supplemental_files.zip › Figure S2_Supplementary Material.pdf]

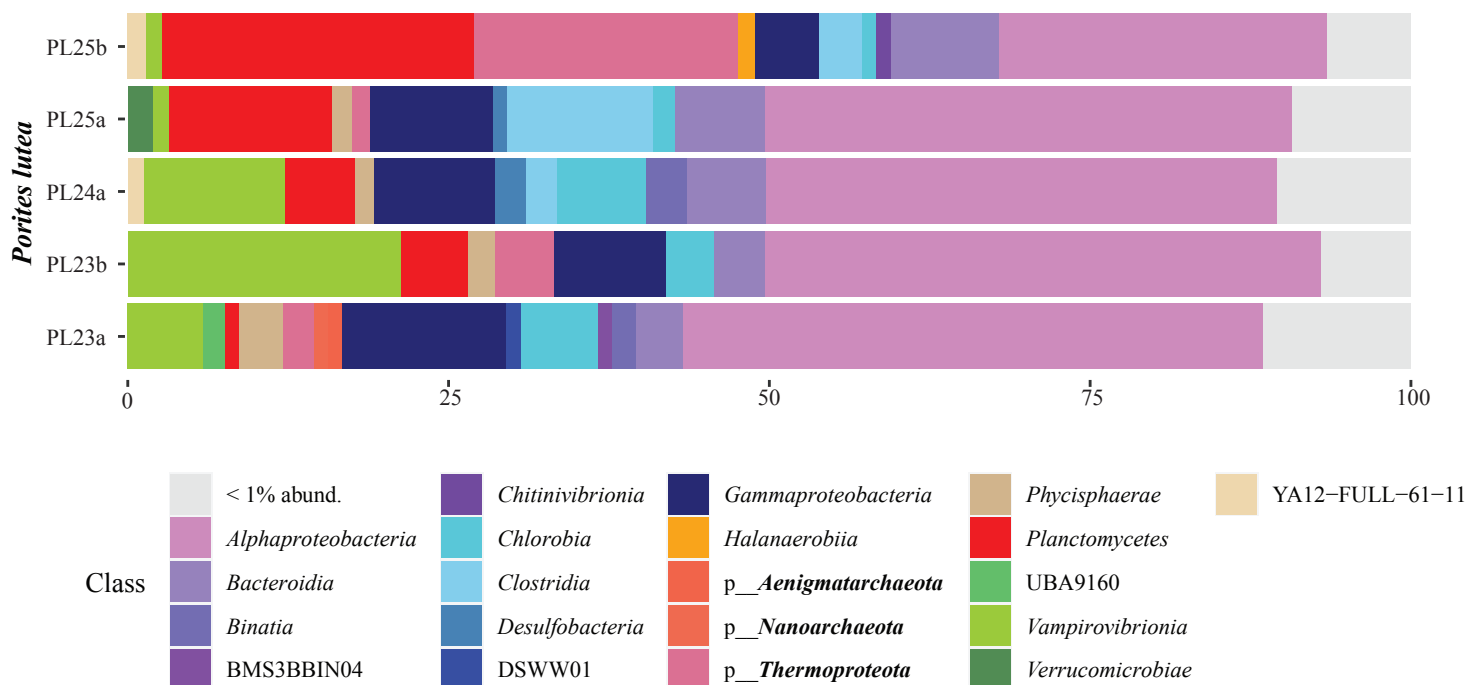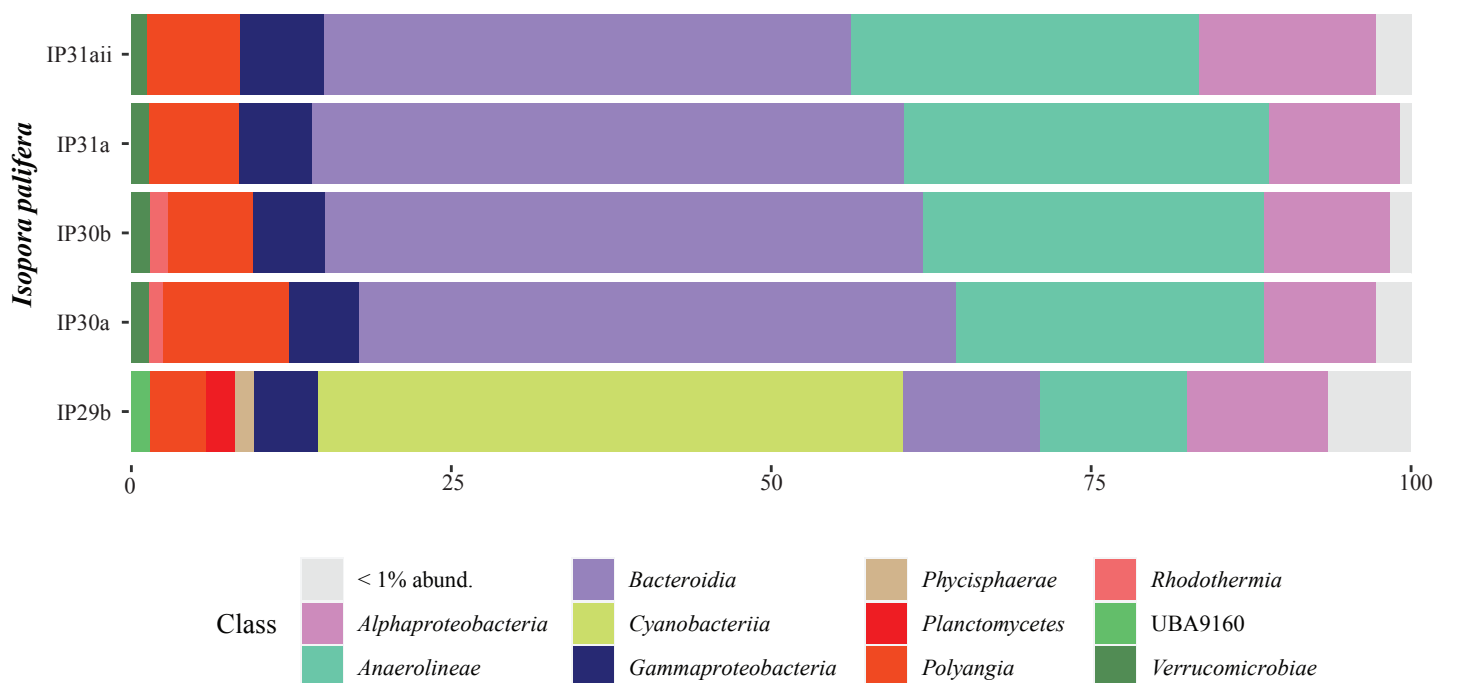

Supplement: giac127_Supplemental_Files [file giac127_supplemental_files.zip › Figure S3_Supplementary Material.pdf]

a

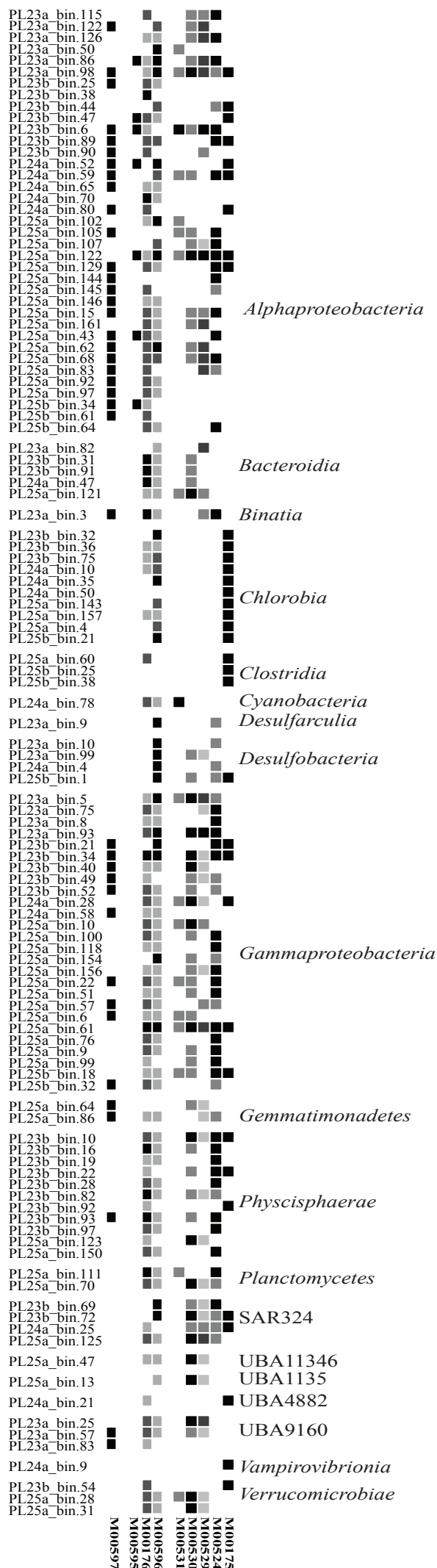

b

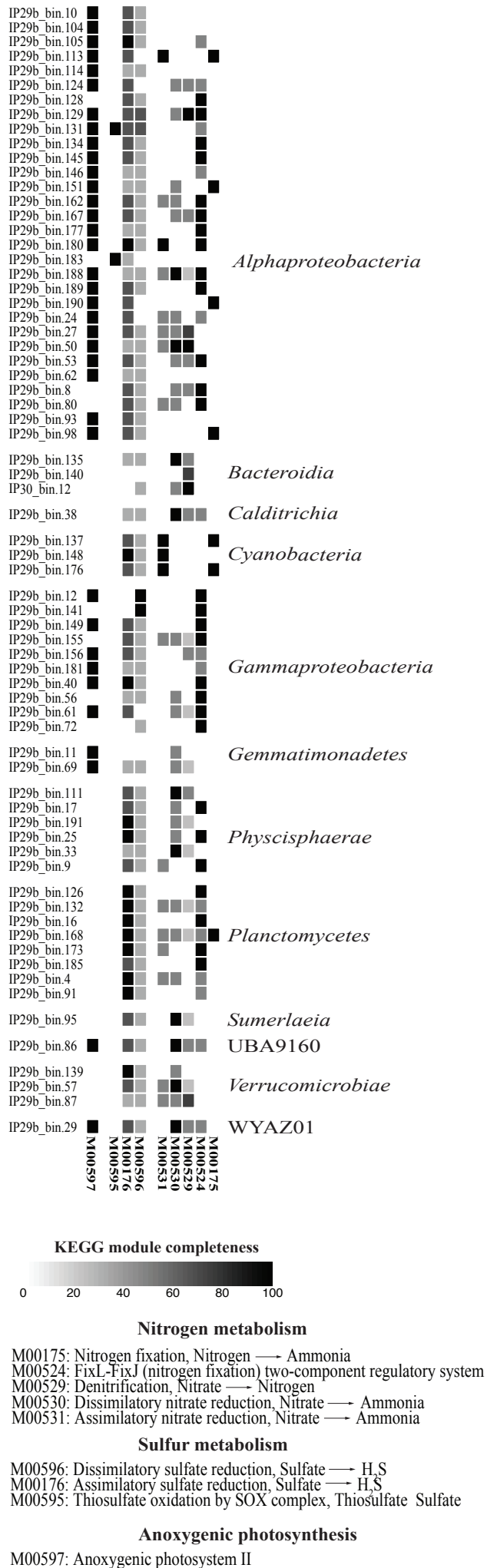

Supplement: giac127_Supplemental_Files [file giac127_supplemental_files.zip › Figure S4_Supplementary Material.pdf]
