# Supplementary material for: Genomic view of the diversity and functional role of archaea and bacteria in the skeleton of the reef-building corals Porites lutea and Isopora palifera
Source: Gigascience. 2023 Jan 23;12:giac127. doi: 10.1093/gigascience/giac127 (PMC9868349; doi:10.1093/gigascience/giac127)
Supplement: giac127_Response_to_Reviewer_Comments_Original_Submission [file giac127_response_to_reviewer_comments_original_submission.pdf]

Author's Response To Reviewer Comments

Close

Point-to-Point response to reviewer comments/suggestions

Reviewer comments/suggestions to Authors
Authors response
Changes in the revised manuscript

Reviewer #1
Reviewer #1: Overall comments:

The article by Tandon et al., describes a comprehensive, high quality genomic characterization of endolithic (skeletal) microbes from two Pacific reef-building corals. The endolithic community is frequently overlooked in coral microbiome studies despite the significant biomass of diverse microbial consortia inhabiting coral skeletons. The study is timely and will be interesting to a broad scientific audience. The language is concise, and the article was very nicely written. I included some specific comments to improve the figures and clarity in the ELP section, but otherwise, I found the manuscript engaging, clear, and easy to follow.

We thank the reviewer for critical reading of the manuscript and suggestions to improve the figures and ELP section. We have addressed these in the revised version manuscript and believe these adequately address the reviewer's comments/suggestions.

Specific comments:

Figures:
Q1. Figure 1 is a great representation of the extensive genomic characterizations included in the study.
A1. Thank you for the appreciation.

Q2. Figures 2 and 3 lack striking visual patterns. Given the complexity of the dataset, I am unsure what to recommend, but I offered some comments to improve general clarity below. Figure 2 would be improved by adding further details to the x-axis. For example, print 'ELP count' below the bar plots, 'ELP categories' below the heat map, and 'MAG phylogeny' beneath the taxon names.
A2. We agree with the reviewer that figures 2 and 3 were difficult to interpret in the first version of the manuscript. Taking reviewer's informative suggestion, we have revised the figures 2 and 3 and they better reflect the meaning we wanted to convey from the analyses.

Q3. Figure 3 was difficult to digest, and I had trouble determining what the reader was meant to take away by analyzing it. Does the purple to pink gradient in the taxonomy column have any significance? Again, labeling the x-axis could help improve clarity (e.g., add an S under the 3 sulfur metabolism columns, and an N under the nitrogen metabolism columns, and maybe a C under the anoxygenic photosystem II).

The main trends I noticed were that several Alphas encoded M00597, and all the Chlorobia in P. lutea fixed nitrogen, and the Plactomycetes in I. palifera reduced sulfate. Otherwise, there was partial module completeness and patchiness among phyla. Is there another way you could illustrate these results conceptually for a graphic in the main text, and shunt this figure to the SI?

A3. We thank the reviewer for this important suggestion. In the revised figure 3, we have only plotted modules which were complete (100%) to showcase the trends more clearly and have moved all the other details in the new figure S4. We believe this improves the readability while preserving the overall information.

Figure Legends:
Q4. Line 820 - What does this 'taxonomic annotation of bacterial MAGs (counterclockwise)' pertain to in the circular graphic? This was very confusing.
A4. Taxonomic annotation of bacterial MAGs (Innermost ring)

Q5. Line 828 - Need a space between lutea | and
A5. Addressed
Q6. Line 831 - Add the full names for HEAT, WD40, and TPR.
A6. Addressed

Results:
Q7. Lines 233-253 - This section came abruptly, and it could help to introduce more rationale for characterizing the different categories of ELPs. I think the idea is very interesting, but I felt lost reading this section. For example, if lines 248-249 were moved earlier in the paragraph, this would help the reader better follow the author's approach. More introduction into the different categories would also help clarity. What are HEAT versus WD40 ELPS (these abbreviations were not defined in the main text)?

A7. Thank you for the suggestion. We have modified this section to reflect the changes and make reading easier to follow.
Line 234: Microorganisms use eukaryotic-like proteins (ELPs) to communicate with their hosts and other eukaryotes.
Line 236-240: MAGs had a broader range of ELPs including, WD or beta-transducin repeats of 40 amino acids often terminating in tryptophan-aspartate dipeptide (WD40), Tetratricopeptide repeat proteins (TPRs), Ankyrin repeat proteins (ARPs), and HEAT repeats, a set of four proteins first identified to contain this repeat motif (Huntington, elongation factor 3, subunit A of phosphatase 2A and signaling kinase TOR1.

Q8. Line 290 - Gammaproteobacteria is very broad. Is it possible to identify the nitrogen-fixer at a finer resolution (genus or family)?
A8. We have added the order names as family and genus were not very informative and most were not annotated. Complete taxonomic annotation for bins is present in the supplementary data file.

Line 288: Interestingly, the oxygen-dependent regulatory nitrogen fixation module (M00524) mediated by FixL-FixJ genes was also complete in several MAGs belonging to Alphaproteobacteria, Gammaproteobacteria (Order: Pseudomonadales, UBA4575, Xanthomonadales, Woeseiales, HTCC5015, DSM-100275 and Chromatiales), Phycisphaerae and Planctomycetes in two coral species (Figure 3b, Supplementary data File).

Q9. Line 366 - Define ARP and TRP.
A9. Corrected

Q10. Line 382 - Do you mean three high quality Chlamydiae MAGs? Revise sentence structure for clarity.
A10. Corrected
Line 392: …three high-quality Chlamydiae MAGs recovered from P. lutea colonies devoted more than 0.2% of their proteome to ARPs (Figure 2a).

Q11. Line 463 - "Coral reefs are net sinks of fixed nitrogen" What do you mean by this? In my experience many coral reefs are net sources of nitrogen, both inorganic and organic forms.
A11. Corrected to sources.

Q1. Reviewer #2: This article presents a metagennomic study on the microbiomes inside the skeleton of two coral species. The authors have clearely stated their purposes and study methods, with clearly explained results. However, it is not clear to me why they "cheery pick" certain proteins or pathways for all downstream analysis. Such approach is not unacceptable, but the authors should provide with a sound explaination why they choose these "functions" for analysis, instead of a overal annotation and enrichment analysis on all possible candidate functions.

A1. We thank the reviewer for critical reading of the manuscript and providing suggestions to improve the

revised version. Main reasons to focus on specific functions ties to the lack of knowledge about these with respect to coral holobiont and especially coral skeleton microbiome, due to lack of genome centric approach. In addition, the coral skeleton microbiom’s functional potential has remained largely unexplored with only marker-based approaches such use of nifH gene for identifying nitrogen fixing ability of the microbes, use of acetylene reduction assays. All these reasons have been highlighted throughout the manuscript

Line 60-67: Endolithic microbes have also been reported to actively participate in nutrient recycling and primary productivity [13,14]. Functional characterisation of complex microbial assemblages in the coral skeleton has mainly relied on selective amplification of target genes, (e.g., nifH [30–32]), experimental approaches such as the acetylene reduction technique, and isotope labelling for probing N2 fixation and other metabolic activities[33,34]. While such studies have yielded important insights into the coral skeleton niche, a genome-centric view of the coral skeletal microbiome and its functional potential remains elusive.

Some minor points:

Q2. In the abstract, 57 microboal class --> 57 class
A2. Addressed
Line 23: spanning 34 phyla and 57 classes.

Q3. Line 38 remove utilisation in
A3. Addressed
Line 37-39: Bacteria have the potential for developing effective assisted evolution strategies such as coral probiotics [4,5] and microbiome manipulation [6] to protect coral reefs.

Q4. Line 46 We need a reference after "coral tissue layer"
A4. Reference has been added.

Q5. Line 119 What is the reason to use a short chain of kmer, longer starting kmer, and shorter ending kmer, as compare to the default setting of MEGAHIT?
A5. We used a longer starting kmer (33) keeping in mind the high biodiversity of the coral skeletal microbiome and to reduce the complexity de Bruijn graph generated during assembly. The ending kmer (99) is same as the default settings of MEGAHIT.

Q6. Line 122 does this mean maxBin2 is not a suitable binner for these samples?
A6. No, maxBin2 only failed to produce bins for just one sample. Therefore, for this specific sample we used Metabat1 as an alternate to keep 3 binning algorithms per sample for consolidating the results. MaxBin2 was able to bin contigs for all the other samples.

Close
