# Supplementary material for: Genomic view of the diversity and functional role of archaea and bacteria in the skeleton of the reef-building corals Porites lutea and Isopora palifera
Source: Gigascience. 2023 Jan 23;12:giac127. doi: 10.1093/gigascience/giac127 (PMC9868349; doi:10.1093/gigascience/giac127)
Supplement: giac127_Reviewer_1_Report_Original_Submission — Linda Wegley Kelly -- 10/3/2022 Reviewed [file giac127_reviewer_1_report_original_submission.pdf]

Reviewer Report

Title: Genomic view of the diversity and functional role of archaea and bacteria in the skeleton of the reef-building corals Porites lutea and Isopora palifera

Version: Original Submission **Date:** 10/3/2022

Reviewer name: Linda Wegley Kelly

Reviewer Comments to Author:

Overall comments:
The article by Tandon et al., describes a comprehensive, high quality genomic characterization of endolithic (skeletal) microbes from two Pacific reef-building corals. The endolithic community is frequently overlooked in coral microbiome studies despite the significant biomass of diverse microbial consortia inhabiting coral skeletons. The study is timely and will be interesting to a broad scientific audience. The language is concise and the article was very nicely written. I included some specific comments to improve the figures and clarity in the ELP section, but otherwise, I found the manuscript engaging, clear, and easy to follow.
Specific comments:
Figures:
Figure 1 is a great representation of the extensive genomic characterizations included in the study.
Figures 2 and 3 lack striking visual patterns. Given the complexity of the dataset, I am unsure what to recommend, but I offered some comments to improve general clarity below.
Figure 2 would be improved by adding further details to the x-axis. For example, print 'ELP count' below the bar plots, 'ELP categories' below the heat map, and 'MAG phylogeny' beneath the taxon names.
Figure 3 was difficult to digest, and I had trouble determining what the reader was meant to take away by analyzing it. Does the purple to pink gradient in the taxonomy column have any significance? Again, labeling the x-axis could help improve clarity (e.g., add an S under the 3 sulfur metabolism columns, and an N under the nitrogen metabolism columns, and maybe a C under the anoxygenic photosystem II). The main trends I noticed were that several Alphas encoded M00597, and all the Chlorobia in P. lutea fixed nitrogen, and the Plactomycetes in I. palifera reduced sulfate. Otherwise, there was partial module completeness and patchiness among phyla. Is there another way you could illustrate these results conceptually for a graphic in the main text, and shunt this figure to the SI?
Figure Legends:
Line 820 - What does this 'taxonomic annotation of bacterial MAGs (counterclockwise)' pertain to in the circular graphic? This was very confusing.
Line 828 - Need a space between lutea | and
Line 831 - Add the full names for HEAT, WD40, and TPR.
Results:
Lines 233-253 - This section came abruptly, and it could help to introduce more rationale for characterizing the different categories of ELPs. I think the idea is very interesting, but I felt lost reading this section. For example, if lines 248-249 were moved earlier in the paragraph, this would help the

reader better follow the author's approach. More introduction into the different categories would also help clarity. What are HEAT versus WD40 ELPS (these abbreviations were not defined in the main text)?
Line 290 - Gammaproteobacteria is very broad. Is it possible to identify the nitrogen-fixer at a finer resolution (genus or family)?
Line 366 - Define ARP and TRP.
Line 382 - Do you mean three high quality Chlamydiae MAGs? Revise sentence structure for clarity.
Line 463 - "Coral reefs are net sinks of fixed nitrogen" What do you mean by this? In my experience many coral reefs are net sources of nitrogen, both inorganic and organic forms.
Very nice work ðŸ˜Š

Methods

Are the methods appropriate to the aims of the study, are they well described, and are necessary controls included? Choose an item.

Conclusions

Are the conclusions adequately supported by the data shown? Choose an item.

Reporting Standards

Does the manuscript adhere to the journal's guidelines on minimum standards of reporting? Choose an item.

Choose an item.

Statistics

Are you able to assess all statistics in the manuscript, including the appropriateness of statistical tests used? Choose an item.

Quality of Written English

Please indicate the quality of language in the manuscript: Choose an item.

Declaration of Competing Interests

Please complete a declaration of competing interests, considering the following questions:

- Have you in the past five years received reimbursements, fees, funding, or salary from an organisation that may in any way gain or lose financially from the publication of this manuscript, either now or in the future?
- Do you hold any stocks or shares in an organisation that may in any way gain or lose financially from the publication of this manuscript, either now or in the future?

- Do you hold or are you currently applying for any patents relating to the content of the manuscript?
- Have you received reimbursements, fees, funding, or salary from an organization that holds or has applied for patents relating to the content of the manuscript?
- Do you have any other financial competing interests?
- Do you have any non-financial competing interests in relation to this paper?

If you can answer no to all of the above, write 'I declare that I have no competing interests' below. If your reply is yes to any, please give details below.

I declare that I have no competing interests

I agree to the open peer review policy of the journal. I understand that my name will be included on my report to the authors and, if the manuscript is accepted for publication, my named report including any attachments I upload will be posted on the website along with the authors' responses. I agree for my report to be made available under an Open Access Creative Commons CC-BY license (http://creativecommons.org/licenses/by/4.0/). I understand that any comments which I do not wish to be included in my named report can be included as confidential comments to the editors, which will not be published.

Choose an item.

To further support our reviewers, we have joined with Publons, where you can gain additional credit to further highlight your hard work (see: https://publons.com/journal/530/gigascience). On publication of this paper, your review will be automatically added to Publons, you can then choose whether or not to claim your Publons credit. I understand this statement.

Yes Choose an item.
