# Supplementary material for: Genomic view of the diversity and functional role of archaea and bacteria in the skeleton of the reef-building corals Porites lutea and Isopora palifera
Source: Gigascience. 2023 Jan 23;12:giac127. doi: 10.1093/gigascience/giac127 (PMC9868349; doi:10.1093/gigascience/giac127)
Supplement: giac127_Reviewer_2_Report_Original_Submission — Zewei Song -- 10/9/2022 Reviewed [file giac127_reviewer_2_report_original_submission.pdf]

**Reviewer Report**

**Title: Genomic view of the diversity and functional role of archaea and bacteria in the skeleton of the reef-building corals Porites lutea and Isopora palifera**

**Version: Original Submission** **Date:** 10/9/2022

**Reviewer name: Zewei Song**

**Reviewer Comments to Author:**

This article presents a metagennomic study on the microbiomes inside the skeleton of two coral species. The authors have clearely stated their purposes and study methods, with clearly explained results. However, it is not clear to me why they "cheery pick" certain proteins or pathways for all downstream analysis. Such approach is not unacceptable, but the authors should provide with a sound explaination why they choose these "functions" for analysis, instead of a overal annotation and enrichment analysis on all possible candidate functions.
Some minor points:
In the abstract, 57 microboal class --> 57 class
Line 38 remove utilisation in
Line 46 We need a reference after "coral tissue layer"
Line 119 What is the reason to use a short chain of kmer, longer starting kmer, and shorter ending kmer, as compare to the default setting of MEGAHIT?
Line 122 does this mean maxBin2 is not a suitable binner for these samples?

**Methods**

Are the methods appropriate to the aims of the study, are they well described, and are necessary controls included? Choose an item.

**Conclusions**

Are the conclusions adequately supported by the data shown? Choose an item.

**Reporting Standards**

Does the manuscript adhere to the journal's guidelines on minimum standards of reporting? Choose an item.

Choose an item.

**Statistics**

Are you able to assess all statistics in the manuscript, including the appropriateness of statistical tests used? Choose an item.

**Quality of Written English**

Please indicate the quality of language in the manuscript: Choose an item.

**Declaration of Competing Interests**

Please complete a declaration of competing interests, considering the following questions:

- Have you in the past five years received reimbursements, fees, funding, or salary from an organisation that may in any way gain or lose financially from the publication of this manuscript, either now or in the future?
- Do you hold any stocks or shares in an organisation that may in any way gain or lose financially from the publication of this manuscript, either now or in the future?
- Do you hold or are you currently applying for any patents relating to the content of the manuscript?
- Have you received reimbursements, fees, funding, or salary from an organization that holds or has applied for patents relating to the content of the manuscript?
- Do you have any other financial competing interests?
- Do you have any non-financial competing interests in relation to this paper?

If you can answer no to all of the above, write 'I declare that I have no competing interests' below. If your reply is yes to any, please give details below.

I declare that I have no competing interests

I agree to the open peer review policy of the journal. I understand that my name will be included on my report to the authors and, if the manuscript is accepted for publication, my named report including any attachments I upload will be posted on the website along with the authors' responses. I agree for my report to be made available under an Open Access Creative Commons CC-BY license (http://creativecommons.org/licenses/by/4.0/). I understand that any comments which I do not wish to be included in my named report can be included as confidential comments to the editors, which will not be published.

Choose an item.

To further support our reviewers, we have joined with Publons, where you can gain additional credit to further highlight your hard work (see: https://publons.com/journal/530/gigascience). On publication of this paper, your review will be automatically added to Publons, you can then choose whether or not to claim your Publons credit. I understand this statement.

Yes Choose an item.
